# Novel Coronin7 interactions with Cdc42 and N-WASP regulate actin organization and Golgi morphology

**DOI:** 10.1038/srep25411

**Published:** 2016-05-04

**Authors:** Kurchi Bhattacharya, Karthic Swaminathan, Vivek S. Peche, Christoph S. Clemen, Philipp Knyphausen, Michael Lammers, Angelika A. Noegel, Raphael H. Rastetter

**Affiliations:** 1 Center for Biochemistry, Medical Faculty, University of Cologne, 50931 Cologne, Germany; 2 Center for Molecular Medicine Cologne (CMMC), University of Cologne, 50931 Cologne, Germany; 3 Cologne Excellence Cluster on Cellular Stress Responses in Aging-Associated Diseases (CECAD), University of Cologne, 50931 Cologne, Germany

## Abstract

The contribution of the actin cytoskeleton to the unique architecture of the Golgi complex is manifold. An important player in this process is Coronin7 (CRN7), a Golgi-resident protein that stabilizes F-actin assembly at the trans-Golgi network (TGN) thereby facilitating anterograde trafficking. Here, we establish that CRN7-mediated association of F-actin with the Golgi apparatus is distinctly modulated via the small Rho GTPase Cdc42 and N-WASP. We identify N-WASP as a novel interaction partner of CRN7 and demonstrate that CRN7 restricts spurious F-actin reorganizations by repressing N-WASP ‘hyperactivity’ upon constitutive Cdc42 activation. Loss of CRN7 leads to increased cellular F-actin content and causes a concomitant disruption of the Golgi structure. CRN7 harbours a Cdc42- and Rac-interactive binding (CRIB) motif in its tandem β-propellers and binds selectively to GDP-bound Cdc42N17 mutant. We speculate that CRN7 can act as a cofactor for active Cdc42 generation. Mutation of CRIB motif residues that abrogate Cdc42 binding to CRN7 also fail to rescue the cellular defects in fibroblasts derived from CRN7 KO mice. Cdc42N17 overexpression partially rescued the KO phenotypes whereas N-WASP overexpression failed to do so. We conclude that CRN7 spatiotemporally influences F-actin organization and Golgi integrity in a Cdc42- and N-WASP-dependent manner.

The actin cytoskeleton is an indispensable machinery of all eukaryotic cells. In the secretory pathway, actin filaments and actin binding proteins (ABPs) are required in organizing the Golgi complex^1^. Spatio-temporal rearrangement of the actin cytoskeleton triggered by the small Rho GTPases of the Ras superfamily dictates actin-driven cellular events. Classically, small GTPases act as molecular switches in the cell by shuttling between GTP-bound (active) and GDP-bound (inactive) state^2^.

Cdc42 GTPase cooperates in actin assembly by inducing filopodia formation and an active Golgi pool of Cdc42 regulates cell polarity and protein trafficking^3^,^4^,^5^,^6^,^7^,^8^. Cdc42 funnels several signals through GTP-dependent binding to effector proteins containing a short stretch of ~15 amino acids referred to as Cdc42/Rac-interactive-binding (CRIB) motif. The core CRIB motif consists of 8 conserved amino acids within the consensus sequence (ISXPXXXFXHXXHVG) and is the minimal effector binding region for Cdc42 and Rac^9^. Mutations of key residues abolish GTPase binding to the effector^10^. However, proteins with partially conserved CRIB motifs can also interact with Rho GTPases in a GTP- or GDP-dependent manner^11^,^12^. Previous reports reveal that N-WASP and Arp2/3 are the downstream effectors of Cdc42 mediating regulation of membrane trafficking at the ER/Golgi interface^13^, but addressing the involvement of ABPs can shed light on the signalling pathway leading to correct Golgi positioning, architecture and trafficking.

Amongst the ABPs present as Golgi-specific isoforms and involved in Golgi structure and function, Coronin7 belonging to the WD40-repeat superfamily is our candidate of interest. In addition to coronins with one WD-repeat domain there exists one long coronin in mammals (Coronin7, CRN7) consisting of tandem β-propellers^14^,^15^. In *Xenopus laevis* coronin a CRIB motif was identified as a potential binding region for GTP-Cdc42 and Rac^16^. A truncated *Xcoronin* led to impaired Rac-mediated spreading and lamellipodia formation. The CRIB motif was later found in a surface accessible loop in several mammalian short coronins between blades 2 and 3 of their β-propeller domain^17^. More recently it was shown that the CRIB motif in the *Dictyostelium discoideum* coronin bound Rac proteins preferentially in their GDP-loaded form and played a prominent role in the regulation of the myosin II cytoskeleton^18^.

So far mammalian CRN7 was known to be involved in Golgi-mediated trafficking^19^,^20^. Yuan and co-workers additionally demonstrated that it promotes F-actin assembly at the TGN and contributes to post-Golgi trafficking^21^. However, the crosstalk between CRN7 and Cdc42, two Golgi-associated proteins^20^,^22^, in regulation of cellular F-actin levels and maintenance of Golgi structure is not yet investigated. We identify semi-CRIB motifs in the tandem β-propellers of CRN7 and analyse their Cdc42 binding activity. Further, we demonstrate that CRN7 loss leads to increased F-actin content and F-actin alterations near the Golgi seems to underlie the observed defects in Golgi integrity. We focus on N-WASP as downstream effector which links Cdc42 activation to Arp2/3-mediated actin polymerization. The activity of N-WASP on Arp2/3 complex is auto-inhibited by an intra-molecular interaction between its B-GBD/CRIB (Basic motif-GTPase binding domain) and VCA (verprolin homology, cofilin homology, and acidic region) domain, which is released upon GTP-Cdc42 binding to the CRIB domain^23^. Our findings uncover a novel interaction of CRN7 with N-WASP, and CRN7 loss is shown to associate with N-WASP overactivity, enhancement in actin-dependent cellular responses and Golgi disruption. Taken together, we propose a signalling mechanism by which CRN7 influences F-actin organization and thereby provides structure to the Golgi apparatus in a Cdc42- and N-WASP-dependent manner.

## Results

### CRN7 is required for structural maintenance of the Golgi complex

**Figure 1:**
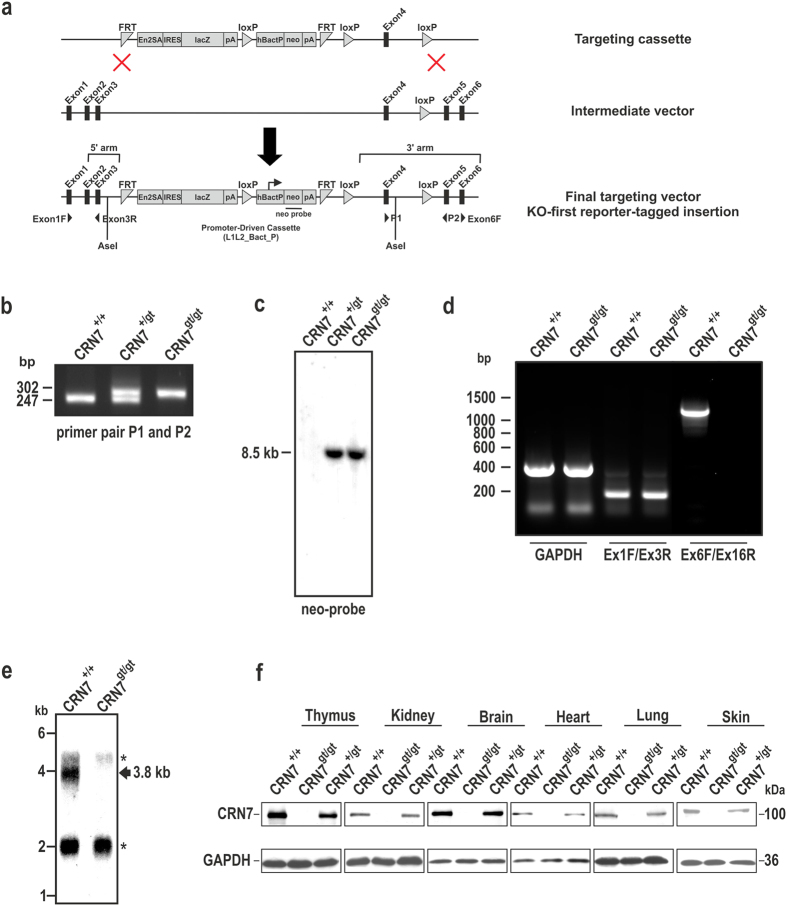
Generation and validation of Coro7^gt/gt^ mice. (**a**) The knockout vector consists of the lacZ gene as a reporter and the neomycin phosphotransferase gene. Genomic locus of the Coro7 gene depicting exons 1, 2, 3, 4, 5 and 6 is illustrated. CRN7 reporter insertion allele: FRT sites, splice acceptor, IRES, lacZ reporter cassette, neomycin selection cassette, 3′ lox P sites flanking the selection cassette and exon 4. The 5′ and 3′ homology arms are derived from the C57BL/6N genetic background. Schematic showing positions of the Southern blot neo probe, AseI restriction enzyme sites, PCR genotyping primers (P1: Ex4F (F-forward), P2: GR7 (R-reverse), arrowheads show direction) and Reverse transcription (RT)-PCR primers (Ex1F-Ex3R and Ex6F, arrowheads show direction; Ex16R is not highlighted here but is further downstream). **(b)** PCR genotyping was performed using tail genomic DNA from wild-type (+/+), heterozygous (+/gt) and homozygous (gt/gt) mice using primers P1 and P2. **(c)** Southern blot analysis of AseI-digested genomic DNA from wild-type, heterozygous and homozygous mice. A radiolabelled probe specific for the neomycin cassette was used for hybridization. **(d)** RT-PCR analysis was done using primer pairs from exon 1 and exon 3 (Ex1F-Ex3R) as well as exon 6 and exon 16 (Ex6F-Ex16R) at the total RNA levels using fibroblasts from wild-type and knockout mice. Control RT-PCR products were generated by a GAPDH-specific primer pair. **(e)** For Northern blot analysis mRNA was isolated from wild-type and knock-out mice fibroblasts and hybridized with a cDNA probe amplified from exon 6 to exon 16. *indicates non-specific binding to 28S and 18S rRNA. **(f)** Immunoblot analysis using lysates from various tissue from wild-type (WT), heterozygous (HET) and homozygous (HOM) mice. Lysates were probed with CRN7-specific mAb K37-142-1.

A Coronin7 knock-out mouse model was generated using the “gene-trap” (gt) approach (Fig. 1a). The floxed exon in this scenario is exon 4, that, when deleted, leads to a frame-shift and a premature stop codon in exon 5. PCR amplification of a sequence stretch including the 3′ distal loxP site allows distinction of wild-type (247 bp), heterozygous (247 bp and 302 bp) and homozygous (302 bp) mice using genomic DNA from tail biopsies (Fig. 1b). Southern-blot analysis further validated the heterozygous and homozygous CRN7 knock-out mice. Digestion with AseI and detection with the radiolabeled neomycin probe shows a band of 8.5 kb for the heterozygous and homozygous mice but no band in the wild-type mice, as expected (Fig. 1c). Semi-quantitative RT-PCR analysis of mRNA isolated from primary fibroblasts of CRN7 wild-type and knock-out mice using a C-terminal primer pair positioned after exon 4 showed that no transcript was expressed from the targeted CRN7 allele but a product of 1024 bp could be amplified in wild-type. However, we could obtain a transcript of 211 bp from both the wild-type and targeted CRN7 allele using an N-terminal primer pair positioned prior to exon 4 (Fig. 1d). We also carried out northern-blot analysis to confirm the mutant and to rule out any possibility of generation of aberrant transcripts. A cDNA probe amplified from the C-terminus of CRN7 and hybridized against total RNA detected a single expected mRNA transcript at 3.8 kb in wild-type mice, whereas no CRN7-specific transcript was detectable in homozygous mutant animal (Fig. 1e). The successful inactivation of the CRN7 gene was confirmed by western-blot analysis where we probed tissue lysates obtained from CRN7 heterozygous and homozygous mice and their wild-type littermates with CRN7-specific monoclonal antibodies. In all tissue lysates from wild-type and heterozygous mice, a signal at ~100 kDa for CRN7 was detectable, but no protein was detected in homogenates from mutant mice (Fig. 1f). The mice were viable and did not show obvious phenotypical, histopathological or behavioural anomalies (unpublished data).

**Figure 2:**
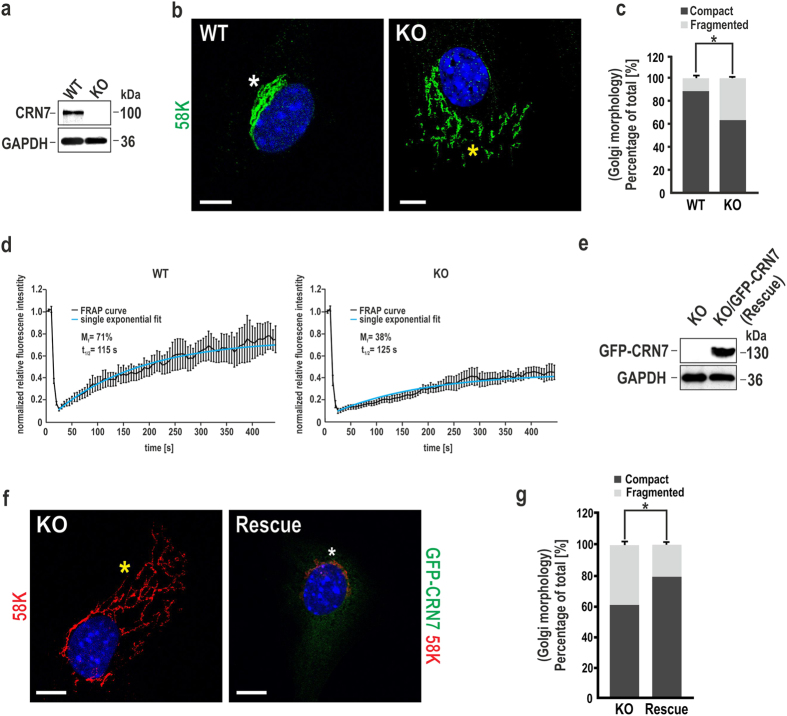
Impact of CRN7 deletion on Golgi architecture. (**a**) Immunoblot analysis of WT and KO fibroblast lysates. CRN7 detected with mAb K37-142-1. GAPDH, loading control. (**b**) Detection of the Golgi apparatus in the fibroblasts using mAb 58 K-9 against the 58K Golgi membrane protein (green); nuclei stained with DAPI (blue). White asterisk, compact Golgi, yellow asterisk, dispersed Golgi. Scale bar = 7.5 μm. **(c)** Analysis of Golgi dispersal for 400 cells. Percentages of WT and KO cells with a fragmented or compact Golgi represented as stacked columns (n = 200 cells each, 2 independent experiments; *P < 0.05). **(d)** WT (left) and KO (right) primary cells transiently transfected with GFP-GalT were photobleached, and FRAP was measured over time. The curves show the normalized FRAP kinetics over time. Fluorescence recovery in bleached areas was monitored every 5 s. Curves of fluorescence intensities were normalized to nonbleached area and the background. Shown is mean ± SEM of 10 cells per condition. M_f_ and t_1/2_ were determined by fitting the curves to a one-phase exponential equation. **(e)** Immunoblot analysis of lysates from KO fibroblasts expressing GFP-CRN7 (rescue). Detection using mAb K37-142-1. **(f)** KO and rescue cells stained for Golgi (red) and rescue cells are green. The Golgi has been marked with asterisks as in (**b**). Scale bar = 10 μm. **(g)** 300 cells scored for their Golgi phenotype. Percentages of WT and KO cells with a fragmented or compact Golgi are illustrated as in (**c**) (n = 150 cells each, 2 independent experiments; *P < 0.05). Except FRAP all data shown as mean ± SD.

Next, we investigated the effect of CRN7 deletion on Golgi architecture in primary dermal fibroblasts isolated from neonatal wild-type (WT) and CRN7 knock-out (KO) mice. Absence of the protein was validated by immunoblotting (Fig. 2a; Fig. S1a). Immunofluorescence analysis with anti-CRN7 antibody validated the localisation of CRN7 at the Golgi region in WT cells but not in KO (Fig. S1b). Staining for 58 K Golgi marker with mAb 58 K-9 revealed a dramatic dispersal of the Golgi ribbon in KO fibroblasts (Fig. 2b). Quantification of images showed that 38% of KO cells had a fragmented Golgi when compared to 12% in case of WT (Fig. 2c). The lack of a stronger penetrance of the effect of knock-out of CRN7 is the fact that CRN7 is not the sole player in this process and likely involves multiple other Golgi proteins/cytoskeletal factors regulating Golgi apparatus morphology. Approximately 62% of WT cells had their Golgi ribbon compact and lying within tens of micrometres from the nucleus. However, in only 34% of KO cells the Golgi ribbon was observed within this range, its juxtanuclear association was lost. Distance between the nucleus and the most distal Golgi ribbon was significantly larger (between 10–30 μm and >30 μm) in randomly selected KO cells (Fig. S1c). Golgi dispersion from the perinuclear position in case of KO can be clearly identified from several cells in Fig. S1d.

We further assessed whether the loss of Golgi ribbon integrity is indeed due to deletion of CRN7 using a quantitative approach based on fluorescence recovery after photobleaching (FRAP) of Golgi-resident enzyme galactosyltransferase fused to GFP (EGFP-GalT). Golgi enzymes diffuse normally through the Golgi ribbon and hence reveal a fast FRAP, however, fragmentation of the Golgi ribbon results in a block in diffusion and hence slow FRAP kinetics as has been previously reported^24^,^25^. WT and CRN7 KO primary cells were transiently transfected with EGFP-GalT, a part of the Golgi membrane was bleached by repeated high intensity laser illumination, and the FRAP of GFP-GalT was examined over time. In Fig. 2d, the normalized FRAP curves represent the mean ± SEM of 10 individual FRAP curves per condition. Calculation of the mobile fraction (M_f_) is also shown in this figure. Compared to WT cells which had the M_f_ value of 71%, the KO cells with fragmented Golgi had a marked decrease of the M_f_ to 38%, indicating that CRN7 is crucial for Golgi structure maintenance.

To investigate whether the observed phenotypes were specifically caused by CRN7 gene inactivation, we reexpressed human (h) wild-type CRN7 tagged with GFP on its N-terminus in CRN7 KO fibroblasts. Human and mouse CRN7 share 86% identity and 91% similarity in their protein sequence, in particular the CRIB domains are highly conserved. The transient expression of GFP-CRN7 in KO cells (Rescue) was confirmed by immunoblotting (Fig. 2e). Since CRN7 itself can localize to the cytosol and the Golgi as has been reported earlier^20^, we examined whether overexpression influences localization as well. We monitored the localization of GFP-CRN7 in our KO fibroblasts and various other cell types (HEK293 and HeLa) (Fig. S2a–d) and observed a variable localization depending on cell type and fixation and permeabilization method employed during immunofluorescence studies. However, the compromised structural integrity of the Golgi complex in KO fibroblasts was restored to normal WT condition in our rescue line (Fig. 2f). Analysis of GFP-CRN7-positive cells indicated that Golgi fragmentation was only observed in a smaller subset of cells (20%) which is close to that observed previously for WT cells (compare Fig. 2g,c; see also Fig. S2e).

### CRN7 deletion influences cell polarization and migration during wound healing

**Figure 3:**
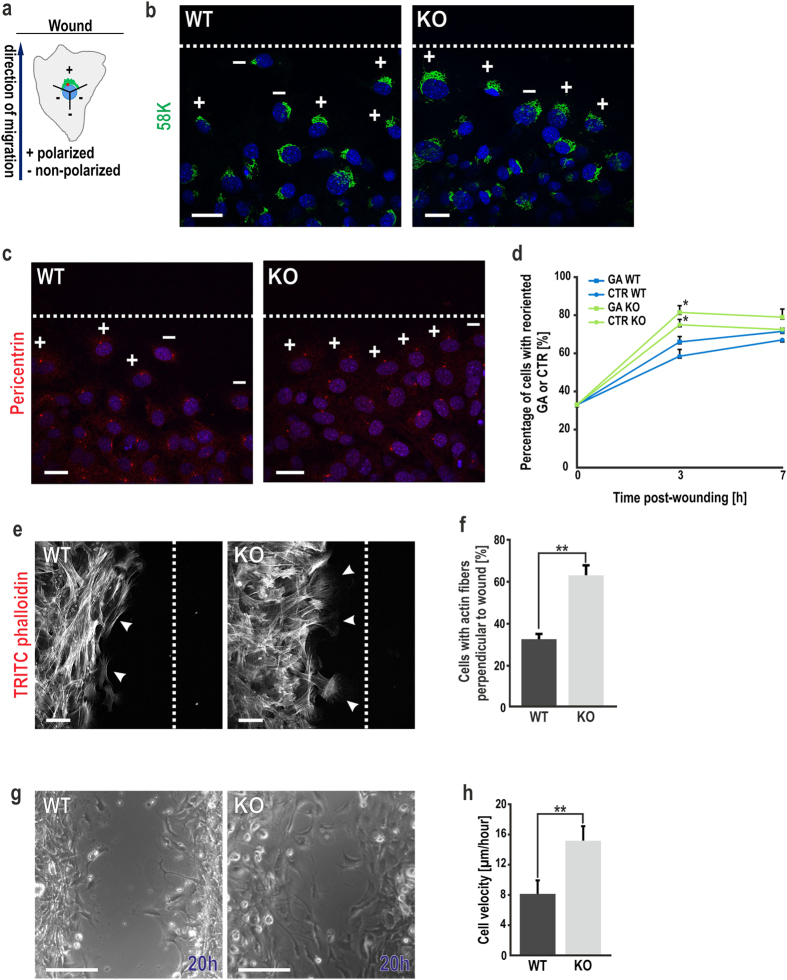
Cell polarization in migrating fibroblasts. (**a**) Schematic showing a cell with Golgi and MTOC positioned within a 120° sector, ( + ) polarized; (−) non-polarized. Blue arrow, direction of migration; black solid line, wound edge. **(b–d)** Scratch-wounded WT and KO fibroblasts stained for (**b**) Golgi (58K, green) and (**c**) centrosome (Pericentrin, cyan) 3 h post-wounding. Nuclei stained with DAPI (blue). The position of the leading zone (broken white line) shown. Scale bar = 25 μm. Line chart (**d**) showing percentage of cells with reoriented Golgi and MTOC (n = 100 cells each time, each condition, 2 independent experiments; *P < 0.05). **(e)** TRITC-phalloidin staining of wounded cell layers (red false-coloured as grey) to visualize the F-actin network. White arrowheads, orientation of the actin fibres (parallel in WT and perpendicular in KO). Scale bar = 50 μm. **(f)** Bar graph with percentage of cells at the wound edge with a polarized distribution of F-actin i.e., perpendicular to leading edge (n = 100 cells each, 2 independent experiments; **P < 0.01). **(g)** Analysis of cell migration over 20 h for WT and KO fibroblasts. Scale bar = 100 μm. Frames from time-lapse phase-contrast videos at the indicated time shown. **(h)** Analysis of the velocity of single cells (in μm/hour). 10 cells from both WT and KO, per position and per edge were considered (n > 200 cells, 5 independent experiments; **P < 0.01). Data shown as mean ± SD.

Centrosome (microtubule organizing centre, MTOC) and Golgi apparatus reorientation towards the leading edge is a prerequisite for collective and directional cell migration during wound healing^26^,^27^. Given that loss of CRN7 directly promotes Golgi stack disassembly, we assayed the ability of our fibroblasts to reorient and migrate into a wound gap. The Golgi apparatus-centrosome (GA-CTR) was counted as polarized when the majority of the staining was located within a 120° sector facing the wound (Fig. 3a). We found that KO cells in the first row could reorganize their Golgi apparatus faster and this was concomitant with MTOC repositioning towards the leading edge, as revealed by immunofluorescence staining of the Golgi (58 K) and MTOC (Pericentrin) in wounded monolayers. Cells in the rear rows were randomly oriented. 66% of WT cells exhibited a polarized Golgi and 58% of them were consistently observed to have polarized their MTOC 3 h post-wounding. In CRN7-null cells the percentage of reoriented Golgi and MTOC was significantly increased to 81% and 75%, respectively (Fig. 3b–d). Finally, although the kinetics of GA and CTR reorientation in WT cells was slower, 7 h after wounding, the percentage of reoriented cells in KO was comparable with respect to control cells (Fig. 3d).

Another hallmark of cell polarization in response to wounding is a polarized actin cytoskeleton localised to the leading edge^28^. We examined the orientation of actin fibres 3 h post-wounding and observed a perpendicular positioning to the wound edge in KO as compared to WT which had most of their actin fibres oriented parallel to the wound (Fig. 3e). Percentage of cells with reoriented actin was 34% in WT, during the same time 65% of KO cells had perpendicularly repositioned their actin (Fig. 3f). To test for the migratory capacity of fibroblasts in a scratch-wound assay, cells were recorded by phase-contrast live microscopy for 20 h post-wounding (Fig. 3g). Single-cell tracking revealed an enhanced mobility for cells lacking CRN7. The mean migration speed of WT cells was 8 μm/h whereas that of KO was 15 μm/h (Fig. 3h) which led to faster gap closure (Fig. S3a). Furthermore, the forward migration index (FMI) and directionality (D) were analysed to understand the relationship between polarity and migration. The analysis revealed a monotonic phenomenon; an unaltered directional persistence resulted in an unchanged FMI in the two cellular conditions (Fig. S3b,c).

No significant difference was observed between WT and KO when quantified for Ki-67-positive cells, Ki-67 strictly being a cell proliferation marker. The percentage of Ki-67-positive cells for both cell types were approximately 30% (Fig. S3d), confirming that the faster wound closure observed in KO is solely due to their migratory properties and is not an additive effect of cell division.

### Loss of CRN7 induces cell spreading and increases F-actin content

**Figure 4:**
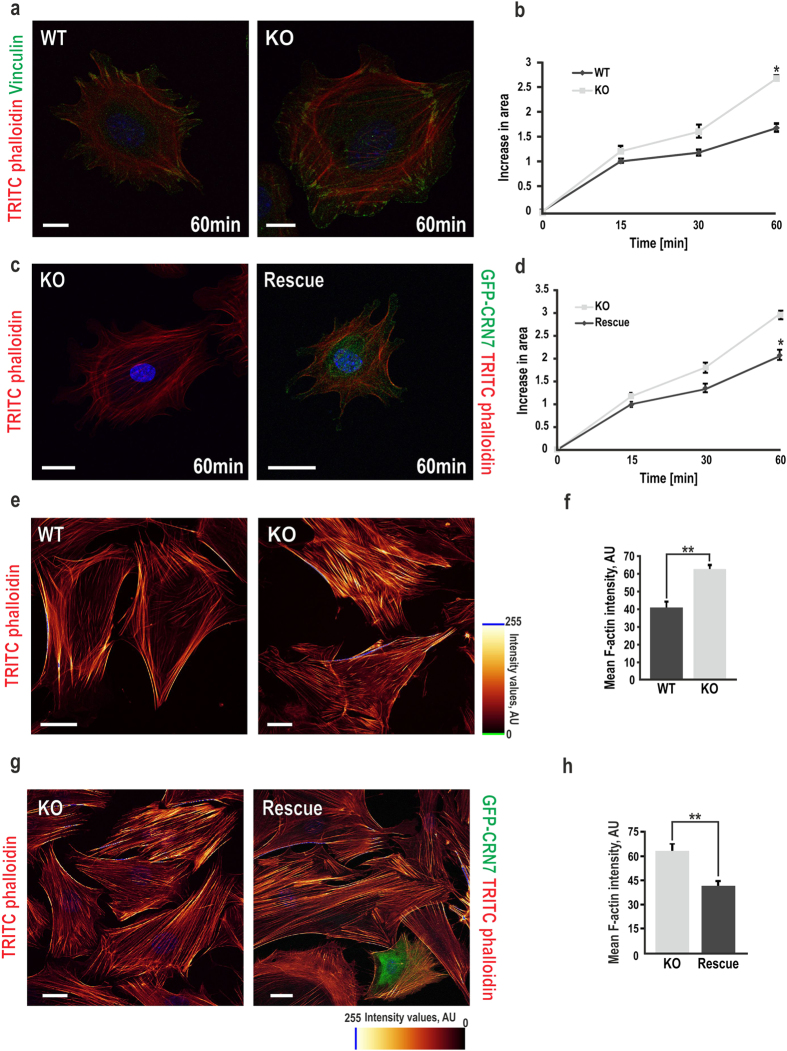
Cell spreading and cellular F-actin content in CRN7 KO fibroblasts. (**a**) Actin cytoskeleton stained with TRITC-phalloidin (red) and focal adhesion stained with vinculin (green) after 60 min of spreading on FN-coated wells analysed by fluorescence microscopy. Scale bar = 10 μm. (**b**) Statistical analysis of (**a**). Line graph shows the increase in projected cell area at different time points (15, 30, 60 min) measured using polygon tool of LAS AF Lite (n = 200 cells each, per time point, 2 independent experiments; *P < 0.05). **(c)** Representative images of KO and rescue fibroblasts (glowing green) stained for F-actin only (red) as in (**a**). Scale bar = 25 μm. **(d)** Fibroblasts quantified for increase in projected cellular area as before (15, 30, 60 min) and represented as line chart (n = 40 cells each, each time point, 2 independent experiments; *P < 0.05). **(e)** Cells fixed and stained as in (**a**) and confocal z-stack images (step size 10) taken. Blue (255), higher actin intensity in the selected channel; yellow to green, decreasing intensity. Scale bar = 25 μm. **(f)** Fluorescence intensity measurement done by a z-stack analysis using confocal microscopy (n = 25 view fields, 3–4 cells per view field, each cell type; **P < 0.01). **(g)** Representative images of fully spread KO and rescue cells (green = GFP) stained for F-actin as in (**e**). Scale bar = 25 μm. **(h)** Fluorescence intensity measurement done as in (**f** ) (n = 40 cells each, 2 independent experiments; **P < 0.01). AU, arbitrary units. Data shown as mean ± SD.

Since increased migration of KO cells can probably be due to their adhesion properties we analysed the attachment of our cells on fibronectin (FN). Strikingly, mutant cells were more adherent than WT and they attached firmly to the FN-coated surface within 30 min of seeding (Fig. S3e). Next, we checked their spreading on FN-coated coverslips. Although the initial cellular area were similar in WT and KO, the surface area of mutant cells increased over the later time-points measured (30 and 60 min), compared to WT (Fig. 4a). This was significant within 1 h of plating and WT cells displayed delayed spreading in this one hour (Fig. 4b). In rescue condition spreading behaviour was reversed to that of WT indicating that CRN7 inhibits cell spreading (Fig. 4c,d). Cell attachment and migration are generally correlated with formation of focal points and actin-rich structures like filopodia and lamellipodia. The focal adhesions though were remarkably increased in KO cells as observed by vinculin staining (Fig. 4a and Fig. S3f), but membrane protrusion formation did not exhibit any significant alterations (Fig. S3g).

The aforementioned phenotypes prompted us to determine the effect of CRN7 deletion on F-actin content. Interestingly, CRN7 loss induced formation of massive F-actin filaments in the fibroblasts as depicted by blue regions in the representative images (Fig. 4e). Quantitative measurements by employing confocal z-stack imaging of TRITC-phalloidin-labelled cells to measure the F-actin content revealed a striking 1.5-fold increase in F-actin intensity in KO fibroblasts when compared to WT (Fig. 4f). The observed increase in F-actin upon CRN7 KO was confirmed through a TRITC-phalloidin based fluorimetric assay where we observed a 1.4-fold increase in mean actin intensity for KO cells when normalized against the DAPI intensity for total cell number and compared with the WT counterpart (Fig. S3h). The mean intensity of the filaments and the F-actin levels in KO fibroblasts were restored upon ectopic expression of GFP-CRN7 (Fig. 4g,h).

We additionally investigated the consequences of increasing cellular F-actin on the structural integrity of the Golgi. We looked into the area around the Golgi and observed that WT cells mostly had low levels of phalloidin staining and no prominent filaments were seen. The Golgi apparatus appeared compact. By contrast, CRN7 KO cells had prominent actin filaments traversing the scattered Golgi stacks, suggesting that F-actin is increased at the Golgi upon CRN7 deletion (Fig. S3i).

### CRN7 interacts with GDP-Cdc42 through its CRIB motifs and regulates downstream cellular processes

**Figure 5:**
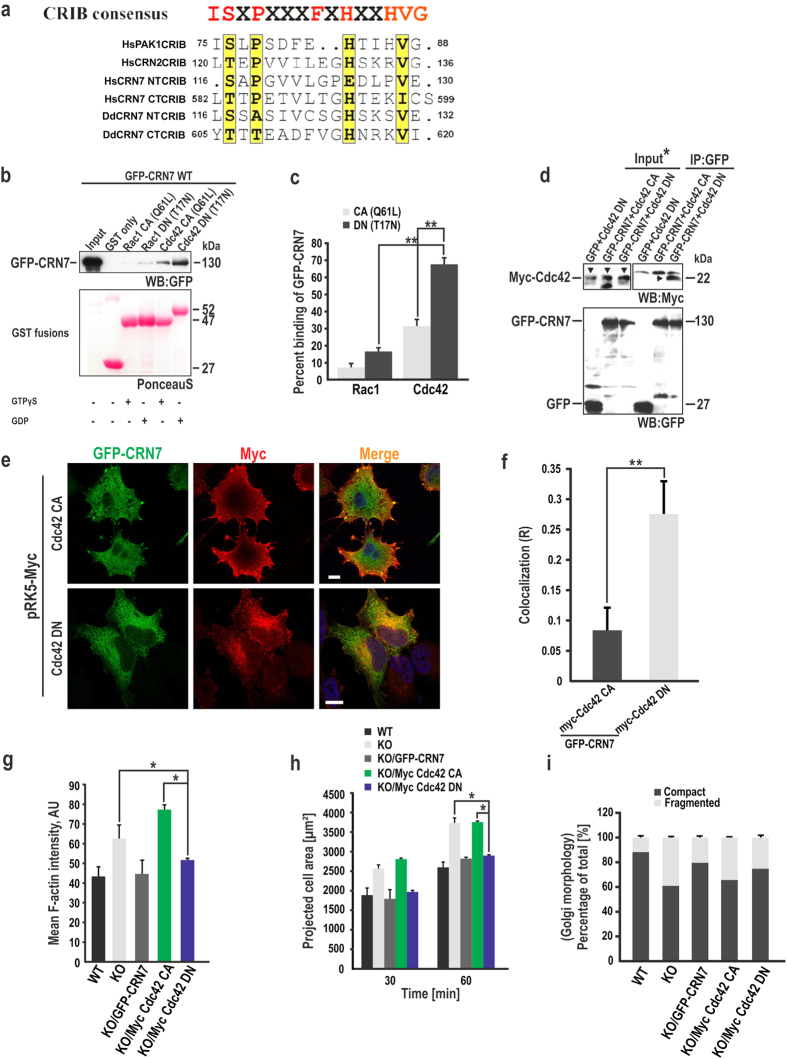
The CRIB motifs in mammalian CRN7 and their impact on the rescue activity of the protein. (**a**) Sequence alignment of human CRN7-CRIB domains (NT and CT) with CRIB domains from human Coronin 1C and D. *discoideum* CRN7. The CRIB consensus shown on top. Similar amino acids boxed in yellow. Highly conserved amino acids marked in red. **(b)** *In vitro* binding assay for full length GFP-CRN7WT with Rac1 and Cdc42 GTPases in their CA/Q61L and DN/T17N forms. Glutathione-Sepharose beads coated with GST alone and GST fusions pre-loaded with GDP (DN) or GTPγS (CA) and incubated with lysates from HEK293T cells expressing GFP-CRN7. PonceauS staining shows the GST fusion proteins. GFP-CRN7 detected with GFP-specific mAb K3-184-2. **(c)** Quantification of GFP-CRN7 bound to Rac and Cdc42 proteins (CA and DN) using ImageJ. Input set at 100% (3 independent experiments; **P < 0.01). **(d)** Lysates from HEK293T cells transiently co-expressing GFP-CRN7 and Myc-tagged Cdc42 GTPase CA and DN forms used for co-IP. GFP-CRN7 pulled down using GFP microbeads. Precipitated Cdc42 detected with anti-Myc mAb 9E10. Immunoprecipitated GFP-CRN7 detected with mAb K3-184-2. GFP used for control. *indicates shorter exposure for the input, arrows point to Myc-Cdc42. **(e)** Co-localisation analysis of CRN7 and Cdc42 mutant proteins. GFP-CRN7 in green; blue, nuclei stained with DAPI, Myc-tagged GTPases, red. Yellow, regions of co-localisation. Scale bar = 10 μm. **(f)** The graph shows a higher CRN7 and Cdc42 DN colocalization coefficient (R) as calculated by Pearson’s correlation coefficient using the ImageJ plugin ‘Colocalization Finder’ (n = 25 cells, unpaired two-tailed t-test, **P < 0.01). **(g)** Mean F-actin intensity values from z-stack images of transfected cells, fixed and stained with phalloidin (n = 10 cells each, 2 independent experiments; *P < 0.05). AU, arbitrary units. **(h)** Quantification of cellular spreading area for transfected cells. Fixed cells stained with phalloidin (n = 12 cells each, each time point, 2 independent experiments; *P < 0.05). **(i)** Percentages of various transfected cells with a fragmented or compact Golgi (n = 32 cells each, 2 independent experiments). Data shown as mean ± SD.

Human CRN7 like CRN7 proteins from other species harbours a CRIB motif in each of its propellers located between blades 2 and 3 at positions 116–130 (NT-CRIB) and 582–599 (CT-CRIB). A comparison with other CRIB motifs shows that they are semi-conserved (Fig. 5a). To probe CRIB-domain interactions with Rho GTPases, we used bacterially-expressed Rac1 and Cdc42 fused to GST in their constitutively active (CA/Q61L) and dominant negative (DN/T17N) forms, preloaded with GTPγS(CA) or GDP(DN), respectively, to precipitate GFP-CRN7 from HEK293T cells. Cdc42 could successfully precipitate GFP-CRN7. Rac1 showed a weaker interaction with CRN7. Notably, the DN fusions of both Rac1 and Cdc42 seemed to have a stronger affinity for GFP-CRN7, with Cdc42DN being more efficient in interacting with CRN7 (Fig. 5b). GST control did not precipitate CRN7 protein. Quantification showed that whereas ~17% of input GFP-CRN7 was bound to Rac1DN and 65% of input was binding to Cdc42DN, only 7% and 30% of input could interact with their CA counterparts, respectively (Fig. 5c). Additionally, we used N-WASP as a positive control and as expected the GTPγS-loaded Cdc42CA very efficiently precipitated N-WASP while GDP-loaded Cdc42DN could not (Fig. S4a). We sought to ascertain the CRN7-Cdc42 interaction *in vivo* and therefore co-expressed Myc-tagged Cdc42CA and DN constructs along with GFP-tagged CRN7 in HEK293T cells to perform co-immunoprecipitation using GFP microbeads. Immunoblotting using Myc antibodies confirmed that CRN7 did co-precipitate Cdc42DN suggesting a complex formation *in vivo* whereas Cdc42CA was not detected in the CRN7 pull down. GFP control did not precipitate Cdc42DN. Probing with GFP-specific mAbs demonstrated successful immunoprecipitation of GFP-CRN7 (Fig. 5d). We further tested a possible co-localisation in immunofluorescence studies of HEK293T cells transiently co-expressing Cdc42 and CRN7. The CA GTPase usually restricted to plasma membrane localisation showed partial co-localisation with GFP-CRN7 at the cell periphery. CRN7 was also found distributed in the cytosol where the DN Rho GTPase localises, and the extent to which it co-localised with the Myc-tagged Cdc42DN near the Golgi was remarkable (Fig. 5e). Furthermore, a quantitative double-immunofluorescence labelling analysis of GFP-CRN7 and myc-tagged Cdc42 mutants revealed a significantly higher colocalization of CRN7 with Cdc42 DN as calculated by the Pearson’s correlation coefficient (R) (Fig. 5f).

In order to analyse the functional consequence of the interaction, Myc-tagged Cdc42CA and DN were expressed in WT and KO fibroblasts (Fig. S4b). Ectopic expression of Cdc42DN in the absence of CRN7 reduced the actin filaments and alleviated the actin content to a considerable extent unlike that of CA overexpression in KO which did not have any impact on the defects (Fig. 5g). The cells were also less spread when compared to KO alone and KO expressing CA mutant (Fig. 5h). Furthermore, overexpression of Cdc42DN partially rescued Golgi morphology in KO cells indicating that the interaction of CRN7 with Cdc42 is necessary for maintaining normal Golgi architecture. Notably, as overexpression of Cdc42CA aggravated the F-actin content in the KO, it had a marked consequence on the morphology of the Golgi complex, which thus remained compromised (Fig. 5i, Table S1).

### CRN7-CRIB motif mutations abrogate Cdc42 binding and have an impact on cellular properties

**Figure 6:**
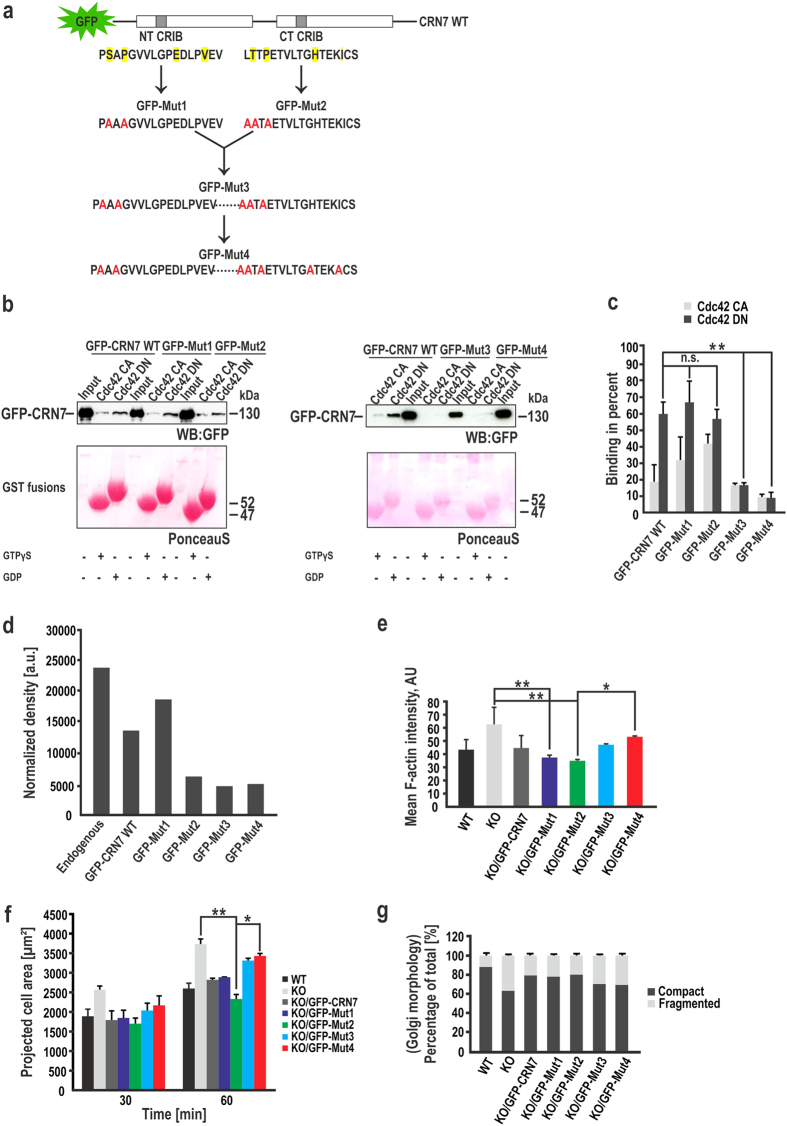
Effect of CRN7-CRIB motif mutations on Cdc42 binding and rescue potential of the proteins. (**a**) Conserved residues in the N- and C-terminal CRIB domains boxed in yellow were mutated to alanine marked in red. The GFP tag is at the N-terminus. (**b**) Binding assay for GFP-CRN7 WT and CRIB mutants (Mut1 and 2, left panel; Mut3 and 4, right panel) with Cdc42 GTPase (CA and DN). Glutathione-Sepharose beads coated with GST fusions, pre-loaded with GDP (DN) or GTPγS (CA) and incubated with lysates from HEK293T cells expressing the CRN7 WT or CRIB mutants. PonceauS staining shows the GST fusion proteins. Probing was with mAb K3-184-2. **(c)** Bar graph showing quantification of GFP-CRN7 WT and CRIB mutants bound to Cdc42 CA and DN using ImageJ. Input set at 100% (3 independent experiments; **P < 0.01 and n.s., not significant). **(d)** Quantification of expression levels of endogenous CRN7 in WT fibroblasts and ectopically expressed GFP-CRN7 WT and CRIB mutants in KO fibroblasts. Cell homogenates from equal numbers of cells were analyzed by western blotting. mAb K37-142-1 detected CRN7 and mAb K3-184-2 recognized the GFP-tagged proteins. GAPDH was used for normalization. **(e)** Mean F-actin intensity values were derived from z-stack images of transfected cells, fixed and stained with phalloidin (n = 15 cells each, 2 independent experiments; *P < 0.05 and **P < 0.01). AU, arbitrary units. **(f)** Quantification of cellular spreading area at 30 and 60 min. Fixed cells stained with phalloidin (n = 25 cells each, each time point, 2 independent experiments; *P < 0.05 and **P < 0.01). **(g)** Percentages of cells under each condition with a fragmented or compact Golgi represented as stacked columns (n = 25 cells each, 2 independent experiments). Data shown as mean ± SD.

To assess the functional significance of CRN7-CRIB motifs we generated CRIB mutants. In general, deletions of the whole motif are normally done; we changed the conserved residues SXP and LTXP in the N-terminal half of NT- and CT-CRIB domains to alanines (A) by site-directed mutagenesis in the plasmid encoding GFP-CRN7WT thereby giving rise to GFP-Mut1 and GFP-Mut2, respectively. GFP-Mut1 and GFP-Mut2 were then combined together in a single construct and termed as GFP-Mut3. Lastly, we designed a GFP-Mut4 by changing the conserved residues HXXXI in the C-terminal half of CT-CRIB to alanines in the plasmid encoding GFP-Mut3 (Fig. 6a). The mutant proteins were expressed in HEK293T cells. In general, endogenous CRN7 was more abundant than the ectopically expressed proteins. GFP-CRN7WT and Mut1 overexpression levels were similar but higher as compared to GFP-Mut2, 3, and 4 levels (Fig. S4c). Immunofluorescence studies in HEK cells revealed that GFP-CRN7WT and all GFP-CRIB mutants shared identical cellular distribution being predominantly present in the cytoplasm but also enriched in a perinuclear region near the Golgi apparatus (Fig. S4d).

Cdc42 binding activity of CRIB mutants were tested using the pull-down assay described above. The WT protein showed a preference for GDP-bound Cdc42 as observed earlier, GFP-Mut1 and 2 behaved like WT, indicating that these point mutations did not alter the binding of CRN7 to Cdc42 (Fig. 6b, left panel). GFP-Mut3 was still able to bind Cdc42, however to a lesser extent and also lost preference for the nucleotide state of Cdc42. Cdc42 binding was nearly abolished in GFP-Mut4 (Fig. 6b, right panel). Upon plotting a graph with the input set as 100%, we observed that 60–70% of the input of CRN7WT, Mut1 and 2 was binding to Cdc42DN, Mut3 lost nucleotide preference rendering 20% of the input to bind equally to Cdc42CA and DN, and only 7–9% of the Mut4 input could bind to Cdc42CA and DN (Fig. 6c). Additionally, we again used anti-N-WASP antibodies to probe the blot from Fig. 6b, left panel, and GTPγS-loaded Cdc42CA very efficiently precipitated N-WASP in all the three reactions unlike GDP-loaded Cdc42DN (Fig. S4e).

Next, the CRIB mutants were expressed in KO fibroblasts and their rescue potential was studied. Examination for their expression levels revealed that in comparison to WT fibroblasts which had highest CRN7 expression levels, GFP-CRN7WT was expressed at slightly lower levels than Mut1 and that of GFP-Mut2, 3, and 4 were markedly lower but were comparable to each other (Fig. 6d). The behaviour of KO fibroblasts expressing GFP-CRN7WT, Mut1 and 2 resembled that of control cells with respect to projection area, F-actin content and Golgi integrity (Fig. 6e–g). Expression of GFP-Mut3 and 4 in KO cells, which are defective in binding to Cdc42, could not sufficiently reconstitute the spreading area and the actin content; instead, the cells behaved similar to KO (Fig. 6e,f). The data suggests that only one intact CRIB motif is necessary and sufficient for CRN7 to regulate these cellular aspects (Table S2). However, these mutants could not re-establish complete Golgi compactness in KO cells and the quantification reflected only a minor reversal towards WT values (Fig. 6g, Table S2) leading us to speculate that this function might depend on the interaction of CRN7 with Cdc42 and is linked to the CRIB domains of CRN7.

### The Cdc42 effector N-WASP is a novel interaction partner of CRN7

**Figure 7:**
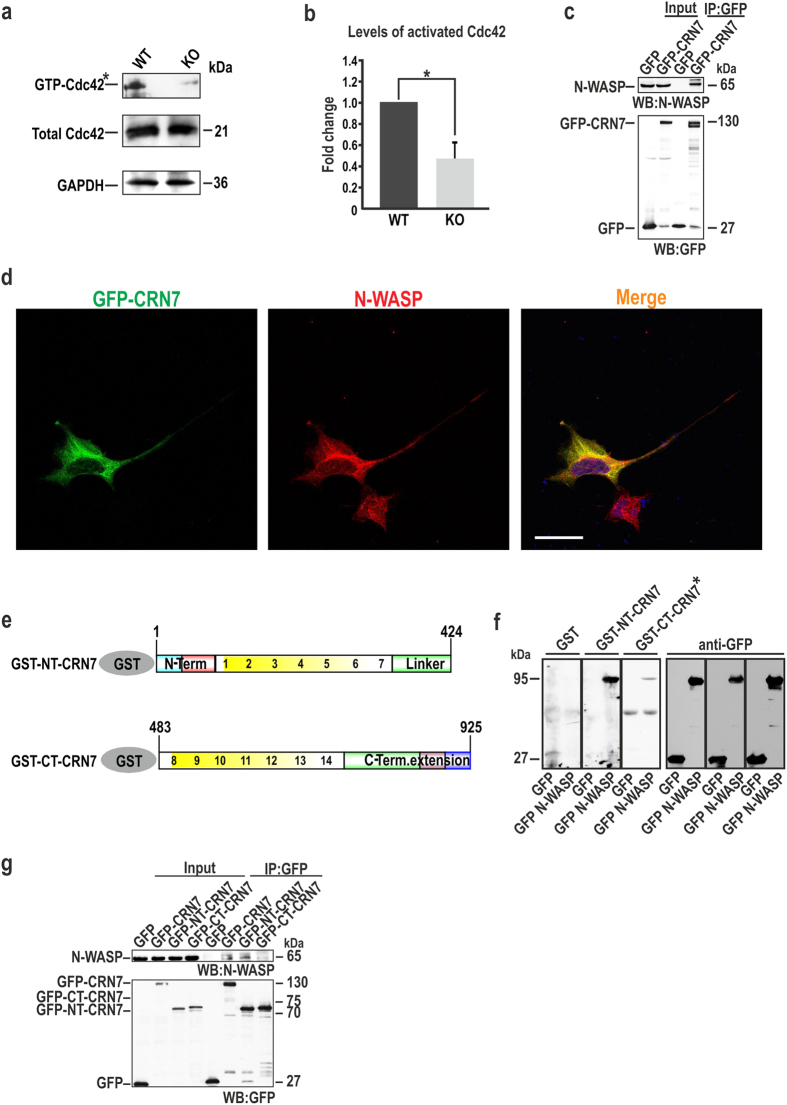
Analysis of N-WASP as a downstream effector. (**a**) GST-Pak1-PBD used to pull down GTP-Cdc42 from fibroblast lysates (upper panel). Lower panel, total Cdc42 levels. Mouse monoclonal Cdc42 antibody detects Cdc42. GAPDH used for normalization. *indicates longer exposure for the pull down and note that the wells loaded with pull down samples from WT and KO were separated by one well in between for visualizing distinct bands. **(b)** Densitometric analysis of active Cdc42 levels. The bar chart shows fold decrease in activated levels as a ratio of the active to the total levels (n = 4 independent experiments, mean ± SD; *P < 0.05). **(c)** GFP microbeads used to precipitate GFP-CRN7 from HEK293T cell extracts, the precipitate probed with rabbit polyclonal anti-N-WASP antibodies. GFP used as control. **(d)** Immunofluorescence analysis of HEK293T cells expressing GFP-CRN7 (green). Staining with N-WASP pAb (red) for endogenous protein. Yellow, merge, co-localisation. Scale bar = 25 μm. **(e)** Schematic showing GST fusion polypeptides of CRN7 (NT and CT). The positions of the amino acids are indicated. **(f)** Affinity-purified GFP-tagged full-length N-WASP blotted to nitrocellulose membranes directly bound by GST-NT-CRN7 and CT-CRN7 but not by GST (left-most panel). *indicates longer exposure of that particular strip of membrane. N-WASP additionally visualized by anti-GFP immunoblotting (whole right panel). **(g)** GFP-CRN7 and its NT and CT fragments precipitated from HEK293T cell extracts using GFP microbeads, N-WASP detected with pAb N-WASP. mAb K3-184-2 verified immunoprecipitated proteins. GFP used as negative control in (**c,f,g**).

Since CRN7 binds to Cdc42 primarily in its GDP-loaded form, we speculate that it might also influence the levels of active Cdc42 in the fibroblasts. The GDIs and GEFs as regulators of the GTPase cycle can bind to GDP-GTPases and lead to their inactivation and activation, respectively. We first examined the levels of active Cdc42 in WT and KO cells in their resting stage by conducting a GST-Pak1-PBD (PBD, CRIB domain of Pak) pull-down assay (Fig. 7a). The Pak1-PBD is derived from rat Pak1 and binds to GTP-Rac1^29^. A PonceauS staining of the membrane shows equal loading (Fig. S5a). The precipitated amounts were compared with amounts of total Cdc42 in the cell lysate normalized against GAPDH (Fig. 7a,b). Quantification of the data revealed that the levels of activated Cdc42 were reduced by a factor of 2 in unstimulated CRN7 KO cells as compared to control cells (Fig. 7b). This data indicates that in unstimulated cells CRN7 deletion prevents the activation of Cdc42, and CRN7 is not a putative GDI. Thereafter, a nucleotide exchange assay was performed using mant-GDP (2′/3′-O-(N-Methyl-anthraniloyl)-guanosine-5′-diphosphate) preloaded on GST-Cdc42WT in presence of molar excess of unlabelled GDP and stimulated with GST-CRN7 or Dbs (Dbl’s big sister), a true GEF^30^. Unlike Dbs which caused a rapid decay in fluorescence, GST-CRN7 was unable to stimulate mant-GDP release from Cdc42 leading to the conclusion that CRN7 does not have a potential GEF activity for Cdc42 (Fig. S5b).

To reconcile our data, we turned our focus towards the most prominent downstream effector of Cdc42, neural WASP (N-WASP) which remodels the actin cytoskeleton by triggering Arp2/3 complex-mediated actin polymerization. It is critical for cell adhesion and motility and the Cdc42/N-WASP/Arp2/3 signalling pathway has been associated with Golgi membranes^13^,^31^. To determine whether CRN7 and N-WASP associate *in vivo*, we carried out immunoprecipitation experiments. GFP microbeads effectively precipitated GFP-CRN7 from HEK293T cell extracts. Immunoblotting analysis of the precipitate with anti-N-WASP antibodies showed that endogenous N-WASP was specifically co-immunoprecipitated with full-length CRN7 (Fig. 7c). We then performed an immunofluorescence analysis with HEK293T cells expressing GFP-CRN7 and observed a prominent cytoplasmic co-localisation of GFP-CRN7 with endogenous N-WASP (Fig. 7d). The next efforts were directed towards mapping the region of CRN7 responsible for interaction with N-WASP and elucidating the nature of their association. To this end, GST-tagged NT- and CT-CRN7 proteins were expressed in bacteria and used in a direct binding assay (Fig. S5c and Fig. 7e). A blot overlay analysis revealed that GST-NT-CRN7 specifically interacted with GFP-N-WASP (Fig. 7f). This demonstrates a direct binding of N-WASP to CRN7 N-terminal region. We also observed an interaction of N-WASP with CRN7 C-terminal region (GST-CT-CRN7), but this interaction was weak and could only be observed upon longer exposure (Fig. 7f). The outcome of an *in vivo* co-IP experiment using CRN7 deletion constructs was identical, wherein GFP-NT-CRN7 could successfully co-immunoprecipitate endogenous N-WASP (Fig. 7g). There might be multiple N-WASP-binding sites in CRN7 but the N-terminus seems to be the major site of interaction.

### N-WASP acts as a mediator of CRN7 functions in actin organization and Golgi structure maintenance

**Figure 8:**
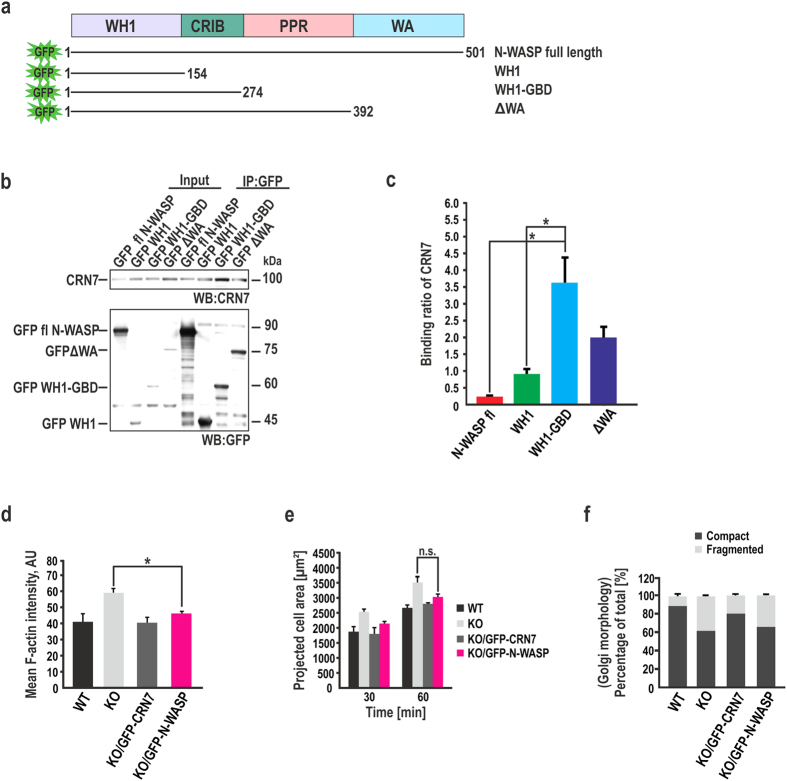
CRN7 regulates cellular processes via N-WASP. (**a**) Domain structure and deletion fragments of N-WASP. The positions of the amino acids are indicated. (**b**) Co-IP using GFP microbeads to precipitate GFP-N-WASP and deletion fragments ΔWA, WH1 and WH1-GBD from HEK293T cell extracts followed by immunoblot analysis using mAb K37-142-1 to detect CRN7. GFP mAb antibody K3-184-2 detected immunoprecipitated N-WASP polypeptides. **(c)** Evaluation of the binding shown in (**b**) represented as a ratio of co-IP (CRN7) to IP (N-WASP fragments) (n = 4 independent experiments, mean ± SD; *P < 0.05). **(d)** F-actin content determined from phalloidin-stained cells. Mean F-actin intensity values derived from z-stack images (n = 20 cells each, 2 independent experiments; *P < 0.05). AU, arbitrary units. Data shown as mean ± SD. **(e)** Quantification of cell spreading area at 30 and 60 min. Cells stained with phalloidin were evaluated (n = 15 cells each, each time point, 2 independent experiments; n.s., not significant). **(f)** Percentages of cells with fragmented or compact Golgi represented as stacked columns (n = 28 cells each, 2 independent experiments).

Considering that N-WASP is a multi-domain protein, it is indispensable to subsequently map the CRN7 interacting region in N-WASP. GFP-tagged N-WASP fragments all containing the WH1 domain (full-length N-WASP, WH1 only, WH1-GBD, and ΔWA) were used to co-precipitate endogenous CRN7 (Fig. 8a). The immunoblot revealed that N-WASP physically associates with CRN7 via the WH1 domain common to all constructs used and hence appears to be necessary and sufficient for the interaction (Fig. 8b). A fragment with the GBD coupled to WH1 (WH1-GBD domain) could however make the interaction stronger. An analysis of binding ratio of the co-immunoprecipitated partner with respect to the immunoprecipitated protein confirmed the findings; WH1-GBD interacted with CRN7 with 4-fold stronger magnitude than WH1 alone. The full-length N-WASP, presumably owing to its auto-inhibitory conformation, showed weak binding affinity towards CRN7 (Fig. 8c).

The functional significance of this interaction was probed by performing rescue experiments with GFP-N-WASP in KO fibroblasts and by investigating whether the CRN7-N-WASP interaction has an activating or inhibiting role in the pathway (Fig. S5d). Ectopic expression of N-WASP in KO fibroblasts could partially alleviate spreading area and F-actin levels suggesting that CRN7 regulates such properties by inhibiting N-WASP-mediated actin polymerization (Fig. 8d,e, Table S3). Additionally, we also examined the Golgi apparatus and found that N-WASP overexpression in KO cells failed to reconstitute the Golgi disruption phenotype and did not differ from that of KO itself (Fig. 8f, Table S3). The loss of CRN7 leads to over activity of N-WASP thus inducing F-actin accumulation and possibly resulting in increased Golgi fragmentation.

## Discussion

The Golgi apparatus in interphase cells is often present as a compact ribbon-like structure that is localised in a juxtanuclear, pericentriolar position. The scenario is very different in mitotic cells where the Golgi disassembles and reassembles as cells go through the cell cycle stages^32^,^33^. This study reports on Golgi complex fragmentation owing to loss of CRN7 using primary fibroblast cells from a CRN7 KO mouse model. The conclusions rest on the evidences gathered from qualitative analysis and quantitative FRAP-based approach. In the current work, we hypothesize that the Golgi protein CRN7 might regulate the activity of the Golgi pool of Cdc42. An earlier report on the spatial control of Cdc42 signalling by Golgi protein GM130 has already implicated the relevance of spatial Cdc42 pools for cell polarity^5^. Future use of FRET biosensors as demonstrated in a study by the Hahn group^4^ to investigate the spatial Cdc42 activity at the Golgi will be highly relevant for understanding the role of CRN7 and Golgi apparatus in directional migration and polarity. Here, we describe a novel pathway in which Golgi-resident CRN7 is possibly able to activate a subset of Cdc42, which in turn regulates Golgi organization in steady-state. Knock-out of CRN7 reduced the levels of active Cdc42. Cdc42 upon activation is known to recruit actin regulator N-WASP to the Golgi apparatus, which is required for assembly of a local actin cytoskeleton that governs morphological changes of the Golgi apparatus^8^,^34^. Therefore, yet another layer of regulation arises as CRN7 is now shown to interact directly with N-WASP and inhibit N-WASP ‘hyperactivity’. CRN7 restricts spurious F-actin events in the cells presumably by this mechanism and thus maintains Golgi integrity.

An increase in F-actin content or pharmacological stabilization of F-actin has earlier been linked to disrupted Golgi structure in case of Formin-like 1 (FMNL1) depletion^35^,^36^. Our WT cells mostly had a pool of short and possibly highly dynamic filaments, which is in accordance with this earlier report while KO cells had strikingly increased actin filaments traversing the Golgi, suggesting that F-actin increases at the Golgi in CRN7-deleted cells. We additionally speculate that elevated F-actin content of KO cells could promote increased actomyosin contractility thereby leading to an increase in cell migration observed here. A similar observation was made for Abl (Abelson murine leukemia)-related gene (Arg/Abl2) kinase where Arg was shown to inhibit F-actin and focal adhesion formation through its regulation of p190RhoGAP. The arg^−/−^ cells migrated faster owing to increased cellular contractility and capability of exerting more force upon their extracellular matrix which could tear focal adhesions from the fibronectin substrate^37^.

In resting conditions CRN7 does not inhibit activation of Cdc42 and this is in variance with results obtained for *Dictyostelium* coronin which was shown to preferentially bind GDP-Rac but coronin mutants had increased basal levels of active Rac as compared to wild type. The authors speculated a GDI-like function for coronin which could prevent Rac from being available for activating downstream effectors by sequestering it^18^. Although a putative GEF domain is absent in CRN7 we hypothesized CRN7 to act as a GEF towards Cdc42, just like RCC1 (Regulator of Chromosome Condensation 1) having a β-propeller which was shown to be a Ran-GEF^38^,^39^. However, a nucleotide exchange assay revealed that CRN7 did not have a GEF activity for Cdc42. A similar situation exists in yeast where Msb3, a member of the TBC/PTM/GYP protein family, selectively binds to GDP-Cdc42, but does not act as GEF or GDI by itself^40^. Is CRN7 an enhancer of a GEF for Cdc42, or does it function as a cofactor for facilitating the interaction of Cdc42 and its GEF to locally activate Cdc42 at the Golgi? It is possible that CRN7 just like Msb3 might simply be involved in capturing and/or maintaining a pool of Cdc42-GDP at the Golgi, poised for activation by a GEF upon external stimuli. We know that Cdc42 functions both at the plasma membrane and at the Golgi, and is required for polarity establishment of the secretory pathway^41^. CRN7 could fulfil this hypothetical role since it itself is involved in anterograde trafficking^19^. Cdc42DN could partially rescue CRN7 KO-induced Golgi abnormality suggesting that Cdc42 is crucial in maintaining Golgi complex architecture.

The relevance of CRN7-CT-CRIB could be due to its surface accessibility and/or the presence of one of the conserved histidines which is known to associate with Asp38 of switch I region of Cdc42^10^. For rescuing the cellular defects in KO, either one of the CRIB domains had to be intact. Where one intact CRIB in Mut1 and 2 was sufficient to modestly rescue the extended Golgi phenotype and re-establish cell spreading area and F-actin intensity in KO, the Mut3 and 4 with defective binding to Cdc42 owing to mutations in both N- and C-terminal CRIB motifs were weaker in preventing disassembly of the Golgi in KO. The results indicated that a CRIB motif interaction with Cdc42 is necessary for CRN7 to maintain proper Golgi structure. Our data is consistent with recently published rescue analysis of *Dictyostelium* long coronin (corB, DdCRN7) CRIB mutants where one functional CRIB could re-establish phagocytosis, cell motility and development^42^.

The report on DdCRN7, the closest homolog of mammalian CRN7, reveals that its interaction with GDP-Rac isoforms regulates actin-driven cellular functions like phagocytosis, development and adhesion via downstream effectors of Rac, namely WASP, SCAR and PAKa, respectively^42^. A similar regulatory cascade can be envisaged for mammalian CRN7 as well by involving members of the Wiskott-Aldrich syndrome protein family N-WASP^43^,^44^. At steady state N-WASP and Arp2/3 are not located at the Golgi membranes. N-WASP mediates the action of Cdc42CA on actin polymerization^45^,^46^. The strong association of N-WASP WH1-GBD domain with NT-CRN7 is worth investigating since GBD-CRIB is also the interface for Cdc42 interaction with N-WASP whereby the auto-inhibitory regulation of N-WASP is released and it is activated. Whether Cdc42 and CRN7 directly compete for the same site to differentially regulate N-WASP remains unresolved, but raises an interesting possibility of multiple ways of regulating N-WASP activity. The data obtained from N-WASP overexpression in CRN7 KO fibroblasts where it fails to rescue the Golgi phenotype implies that N-WASP is ‘hyperactive’ in KO. We propose a provisional model in which CRN7 spatio-temporally regulates active Cdc42 binding to N-WASP or alternatively CRN7 itself directly interacts with and inhibits N-WASP activity, resulting in a novel mechanism by which the ability of CRN7 to coordinate F-actin dynamics is crucial for maintaining Golgi architecture (Fig. S6). Further studies that elucidate how CRN7 as a Golgi-resident actin regulator affects the N-WASP-triggered actin polymerization and later its depolymerisation in the presence or absence of Cdc42 will be of great interest for the field.

## Materials and Methods

### Coronin7 knock-out mice

The murine Coro7 gene located on chromosome 16 (genomic contig NC_000082.6) comprises of 28 exons. A CRN7 knock-out vector targeting the CRN7 gene was constructed and made available by European Conditional Mouse Mutagenesis consortium (EUCOMM), Helmholtz Zentrum München, Munich, Germany (http://www.mousephenotype.org/about-ikmc/eucomm). EUCOMM uses a promoter-driven targeting cassette and recombination of the targeting vector with the CRN7 gene resulted in the “knock-out-first reporter-tagged insertion allele”^47^. Clone BO4 was obtained and the ES cells were microinjected into blastocysts at the Biocenter Oulu, Transgenic core facility, University of Oulu, Finland (http://www.oulu.fi/biocenter/tg-core). Injected blastocysts were subsequently transplanted into the uterus of pseudo-pregnant mice. Chimeric offspring born were bred in C57BL/6N background for germ line transmission. The mice were now heterozygous for the CRN7 reporter insertion allele. They were of pure C57BL/6N genetic background, and could be bred to homozygosity. Animals were housed in the facility of the Center for Molecular Medicine Cologne (CMMC), University of Cologne. All animal protocols were approved by the local veterinary authorities. All experiments were performed according to institutional guidelines and an animal license of the State Office of North Rhine-Westphalia, Germany.

### Cell culture, primary fibroblast isolation and transfection

Adherent mammalian cells were cultured in DMEM (PAN Biotech) supplemented with 10% fetal calf serum and antibiotics.

Neonatal animals were used in our studies; age and sex-matched littermates were used as controls. New born mice (day 1 old) were decapitated and the whole skin of the torso was isolated. Dispase II (5 mg/ml) incubation of the skin overnight at 4 °C separated the dermis from epidermis. The isolated dermis was digested with Collagenase I (400 U/ml) for 1 h at 37 °C. The cells obtained were used between passages 2–8 for our experiments.

Mouse primary dermal fibroblasts were transfected as per the protocol of Amaxa Nucleofection kit (Lonza). For HEK293T cells, PEI transfection reagent (Sigma) (1 μg/μl) was used with DNA in a 3:1 ratio of PEI to DNA. Transfected cells were harvested after 24–48 h.

### Immunofluorescence staining and confocal imaging

Immunofluorescence analyses were performed as described earlier^42^,^48^. PBS washed cells grown on 12 mm fibronectin-coated (20 μg/ml) or non-coated coverslips were fixed with 4% paraformaldehyde or ice-cold methanol and permeabilized using saponin or Triton X-100 as required. The primary antibodies (see supplementary materials) were diluted as recommended in 1X PBG (PBS, BSA, and Fish Gelatin). Appropriate secondary antibodies (coupled to AlexaFluor 488/568/647) were also diluted in 1X PBG; nuclei were stained with DAPI and F-actin with TRITC-phalloidin. All microscopic images were acquired with a TCS SP5 confocal laser scanning microscope (Leica, software LAS AF version 2.6.0.7266), unless stated otherwise.

### Golgi FRAP analysis

To investigate Golgi membrane continuity, we transiently transfected EGFP-GalT in our primary fibroblasts grown on ibidi glass-bottom dishes. After 24 h post-transfection the diffusion mobility of GFP-GalT protein in living cells was imaged at 37 °C using a laser scanning confocal microscope. Settings for FRAP analysis were as follows: objective HCX PL APO lambda blue 63.0 × 1.40 OIL UV, 1024 × 512 pixel resolution, bi-directional x-line scanning at 1,000 Hz, pinhole 280 μm, photobleaching at fly mode with 100% laser light intensities (405, 458, 476, 488 nm), image recording at 495 nm-550 nm for GFP with 2-line average. 3 images were acquired before 3 bleaching iterations diminished the fluorescence signals within the ROI (region of interest; 2 × 2 μm). Recovery of fluorescence was observed for 84 subsequent frames by scanning every 5 s. Raw data (ROI) first were background corrected followed by a correction of photobleaching of the ROI values using the unbleached Golgi region intensity at each time point, and then normalized to the pre-bleach intensity. Resulting values were used to calculate the mobile fraction (M_f_) and half-life times of recovery (t_½_) via single-exponential curve fitting (solver add-in of Microsoft Excel).

### *In vitro* cellular assays

Cells seeded on coverslips pre-coated with fibronectin were allowed to adhere and spread for various time points followed by fixation and phalloidin staining. Projected cell area was evaluated using LAS AF Lite software. Membrane protrusion formation was quantified using ImageJ.

Wound healing or cell migration assays were conducted as described previously^48^. Golgi apparatus-centrosome reorientation or actin polarization assays were performed on cells grown in ibidi culture inserts over coverslips. Cells migrating into the wound gap were fixed and stained for Golgi (GA), centrosome (CTR) and actin.

TRITC-phalloidin-labelled cells were assessed for the relative fluorescence intensity using the TECAN plate-reader (infinite M1000). Alternatively, confocal z-stacks were acquired for fixed and stained cells and mean fluorescence intensity was evaluated using the LAS AF Lite software.

### Generation of fusion proteins and expression and purification of proteins in bacterial and mammalian cells

GFP-CRN7 WT and CRIB mutants (GFP-Mut1, 2, 3, 4) were transfected into HEK293T or fibroblast cells as described above. Cell lysates were prepared using ice cold lysis buffer as described^42^.

For the generation of different CRN7 deletion constructs as GST-fusion proteins, appropriate coding sequences were PCR amplified and cloned into the expression vector pGEX-4T-1 using EcoRI and XhoI sites. GST-Cdc42 or CRN7 mutants were expressed in *E. coli* BL21 and purified from the soluble fraction using Glutathione Sepharose affinity columns (GE Healthcare). Elution buffer (20 mM Glutathione, 50 mM Tris-HCl, pH 8.0, 0.3% Sarkosyl) was used to elute GST-tagged protein from beads as per the requirement of the experiment.

### Pull-down, co-immunoprecipitation and blot overlay experiments

CRN7-Cdc42 interaction assays were conducted as described earlier^42^.

GST-Pak1-PBD pull down assay was performed as described previously^18^,^29^. Western blot analysis was done with mouse mAb against Cdc42 and the amount of activated Cdc42 was quantified using ImageJ.

GFP- or Myc-antibody pre-coated microbeads (μ-MACS, Miltenyi Biotech) were used for IP or co-IP experiments as described by the supplier. Alternatively, Protein-A Sepharose beads coated with GFP-specific polyclonal antibodies were incubated with cell lysates overexpressing a GFP-tagged protein for IP.

For overlay assays, membranes carrying the desired proteins were incubated with 5 μg/ml of eluted GST or GST fusion proteins (diluted in PBS, 0.1% Tween-20). Binding of GST-CRN7 deletion proteins to GFP-N-WASP was detected by incubation with GST-specific polyclonal antibodies. The blot was later probed with GFP-specific antibodies.

### Data analysis, image processing, and statistical evaluation

Images were processed using ImageJ 1.47d (NIH), Adobe Photoshop CS version 8.0, and figures assembled using CorelDraw Graphics Suite X4. The model was generated using Pathway Builder Tool (V2.0). Data analyses and statistical evaluations were carried out using Microsoft Excel; the number of independent experiments, standard deviations/errors, and p-values (Student’s t-test) are indicated in the figure legends.

## Additional Information

**How to cite this article**: Bhattacharya, K. *et al.* Novel Coronin7 interactions with Cdc42 and N-WASP regulate actin organization and Golgi morphology. *Sci. Rep.* **6**, 25411; doi: 10.1038/srep25411 (2016).

## Supplementary Material

Supplementary Information
