# Supplementary material for: Novel Coronin7 interactions with Cdc42 and N-WASP regulate actin organization and Golgi morphology
Source: Sci Rep. 2016 May 4;6:25411. doi: 10.1038/srep25411 (PMC4855144; doi:10.1038/srep25411)
Supplement: Supplementary Information [file srep25411-s1.pdf]

Novel Coronin7 interactions with Cdc42 and N-WASP regulate actin organization and Golgi morphology

Kurchi Bhattacharya[1,2,3], Karthic Swaminathan[1,2,3#], Vivek Peche[1,2,3], Christoph S. Clemen[1,2,3], Philipp Knyphausen[3], Michael Lammers[3], Angelika A. Noegel[1, 2, 3*], Raphael H. Rastetter[1,2,3]

[1]Center for Biochemistry, Medical Faculty, [2]Center for Molecular Medicine Cologne (CMMC) and [3]Cologne Excellence Cluster on Cellular Stress Responses in Aging-Associated Diseases (CECAD), University of Cologne, 50931 Cologne, Germany

*corresponding author
Dr. Angelika A. Noegel
Institute of Biochemistry I, Medical Faculty
University of Cologne
Joseph-Stelzmann-Str. 52
50931 Cologne
Tel: 0049 221 478 6980
Fax: 0049 221 478 6979
Email: noegel@uni-koeln.de

[#]present address
Cancer Research UK, Beatson Institute, Glasgow

Supplementary information

Figure legends

Figure S1. Further characterisation of Golgi structure in primary fibroblasts.

(a) A full anti-CRN7 blot with fibroblast cell lysates prepared from 3 different mice each of WT and KO. CRN7 detected with mAb K37-142-1. The strongly labelled band at ~55 kDa is non-specific. **(b)** CRN7 localization in the WT cells at the Golgi. CRN7 (green) detected with mAb K37-142-1. DAPI stains nuclei (blue). Absence of the protein in KO cells. Scale bar = 25 µm. **(c)** Percentages of WT and KO cells having nucleus-to-Golgi distance of <10, 10-30, and >30 µm determined using 58K marker (n = 200 cells each, 2 independent experiments; *, $P < 0.05$). **(d)** Overview of multiple WT and KO cells showing their respective Golgi morphologies. Detection using 58K (green). DAPI stains nuclei (blue). Scale bar = 25 µm.

Figure S2. Localization of GFP-CRN7 in various cell types.

(a) GFP-CRN7 localizes to the Golgi region in KO primary cells and partially colocalizes with the Golgi marker 58K when fixed with PFA and permeabilized with saponin only. Scale bar = 25 and 10 µm respectively. **(b)** HeLa cells fixed and permeabilized with Methanol shows perinuclear GFP-CRN7 accumulation and cytoplasmic staining. Scale bar = 10 µm. **(c)** HEK293 cells fixed with PFA and permeabilized with saponin shows GFP-CRN7 at the Golgi and this colocalizes with Crn7 mouse mAb staining. Scale bar = 5 µm. **(d)** GFP-CRN7 is predominantly cytosolic in HEK293 cells fixed with PFA and permeabilized with Triton X-100. Scale bar = 10 µm. **(e)** Overview of multiple KO and rescue cells (GFP-CRN7 in KO, green) stained for Golgi (red). Scale bar = 25 µm.

Figure S3. Additional characterisation of actin phenotypes in primary fibroblasts.

(a) Remaining gap after 20 h of wounding analyzed in terms of pixel number using Magnetic Lasso tool. Closed gap area shown in terms of µm^2/hour (1 pixel = 0.72 µm, n = 5 independent experiments done in quadruplicates; **, $P < 0.01$). **(b-c)** Cell trajectory data are used to evaluate the Directionality, D (euclidean distance/accumulated distance) and Forward Migration Index towards the y-axis, FMI, for primary fibroblasts using ImageJ 'Manual Tracking and Chemotaxis' tool (n = 12 cells, 2 independent experiments; n.s., not significant). **(d)** Proliferation rate of fibroblasts determined with mAb against Ki-67, DAPI stains nuclei. Ki-67-positive cells represented in percentage (n = 250 cells each; n.s., not significant). **(e)** Cell adhesion of fibroblasts determined using Neutral Red dye (t = 30 min, n = 3 independent experiments done in quadruplicates; *, $P < 0.05$). **(f)** Cells stained with mouse monoclonal vinculin were counted for focal adhesion number using the ImageJ 'Particle Analyzer' tool (n = 36 cells; 2 independent experiments; *, $P < 0.05$). **(g)** Membrane protrusions classified from TRITC-phalloidin stained cells. Proportion of lamellipodia to filopodia shown in a stacked column chart (n = 100 cells each). **(h)** Cells grown on FN-coated 6-well plates fixed and stained with TRITC-phalloidin, normalized against DAPI for cell number. Fluorescence intensity measured using TECAN reader by selecting multiple points in the wells to have a cumulative intensity read-out of F-actin content. (n = 4 independent experiments done in triplicates; **, $P < 0.01$). RFU, relative fluorescence unit. Data shown as mean ± SD. **(i)** z-stack images of a magnified area (yellow square) around the Golgi (58K, green) in WT and KO cells stained with TRITC-phalloidin (F-actin, red) and DAPI (nuclei, blue). Scale bar = 25 µm for whole cell view.

Figure S4. Further analysis of CRN7-CRIB and Cdc42 interactions.

(a) Positive control blot. In vitro binding assay for full length GFP-CRN7WT with Rac1 and Cdc42 GTPases in their CA/Q61L and DN/T17N forms. Glutathione-Sepharose beads coated with GST alone and GST fusions, pre-loaded with GDP (DN) or GTPγS (CA) and incubated

with lysates from HEK293T cells expressing GFP-CRN7. N-WASP in input and Cdc42 CA pull-down detected with a rabbit pAb. **(b)** Expression levels of Myc-Cdc42 CA and DN mutants in fibroblasts as detected with mAb 9E10. GAPDH, loading control. **(c)** Densitometric quantification of expression levels of endogenous CRN7 and ectopically expressed GFP-CRN7 WT and CRIB mutants in HEK293T cells. Cell homogenates from equal numbers of cells analysed by western blotting. mAb K37-142-1; CRN7 and mAb K3-184-2; GFP-tagged proteins. GAPDH used for normalization. **(d)** Localization of the proteins in HEK293T cells. Cells fixed and stained with mAb specific for Golgi marker GM130 (red), nuclei stained with DAPI (blue). Scale bar = 5 µm. Yellow, merge, co-localization of overexpressed GFP-CRN7 WT or CRIB mutants with GM130. **(e)** Positive control blot. Binding assay for GFP-CRN7 WT and CRIB mutants (Mut1 and 2) with Cdc42 GTPase (CA and DN) as in (a). N-WASP in input and Cdc42 CA pull-downs detected with a rabbit pAb.

Figure S5. Identifying the nature of interaction of CRN7 with Cdc42 and N-WASP.
(a) PonceauS staining for the full blot of Pak1-PBD pull-down assay. **(b)** GST-Cdc42 WT preloaded with the fluorescent GDP analogue mant-GDP was incubated in buffer containing molar excess of unlabelled GDP and stimulated with GST alone, GST-CRN7 and Dbs. Release of GDP was monitored by a loss of fluorescent signal over the indicated time points. RFU, relative fluorescence unit. **(c)** Coomassie stained gel showing the CRN7 deletion constructs (NT and CT). **(d)** Expression levels of GFP-tagged full-length N-WASP in fibroblasts. Probing was with mAb K3-184-2. GAPDH, loading control.

Figure S6. Provisional model for CRN7-Cdc42-N-WASP association.
In WT (left), CRN7 might act to positively facilitate Cdc42 activation leading to N-WASP activation downstream in the pathway. Cdc42 in its GTP bound form can recruit N-WASP to the Golgi complex and trigger actin polymerization. As a checkpoint, CRN7 directly binds N-

WASP and competitively (?) inhibits its activity upon constitutive Cdc42 activation to restrict spurious F-actin content. However, in KO (right), any Cdc42-GEF can still activate Cdc42 and lead to N-WASP “hyperactivity” (in the absence of inhibition by CRN7) which then promotes F-actin formation leading to an enhancement of actin-driven processes. In WT, controlled F-actin levels at the Golgi helps to maintain its structure, while in KO, increased F-actin content is associated with disrupted Golgi integrity. Thus, CRN7 regulates actin organization and Golgi architecture via Cdc42 and N-WASP.

Figure S1

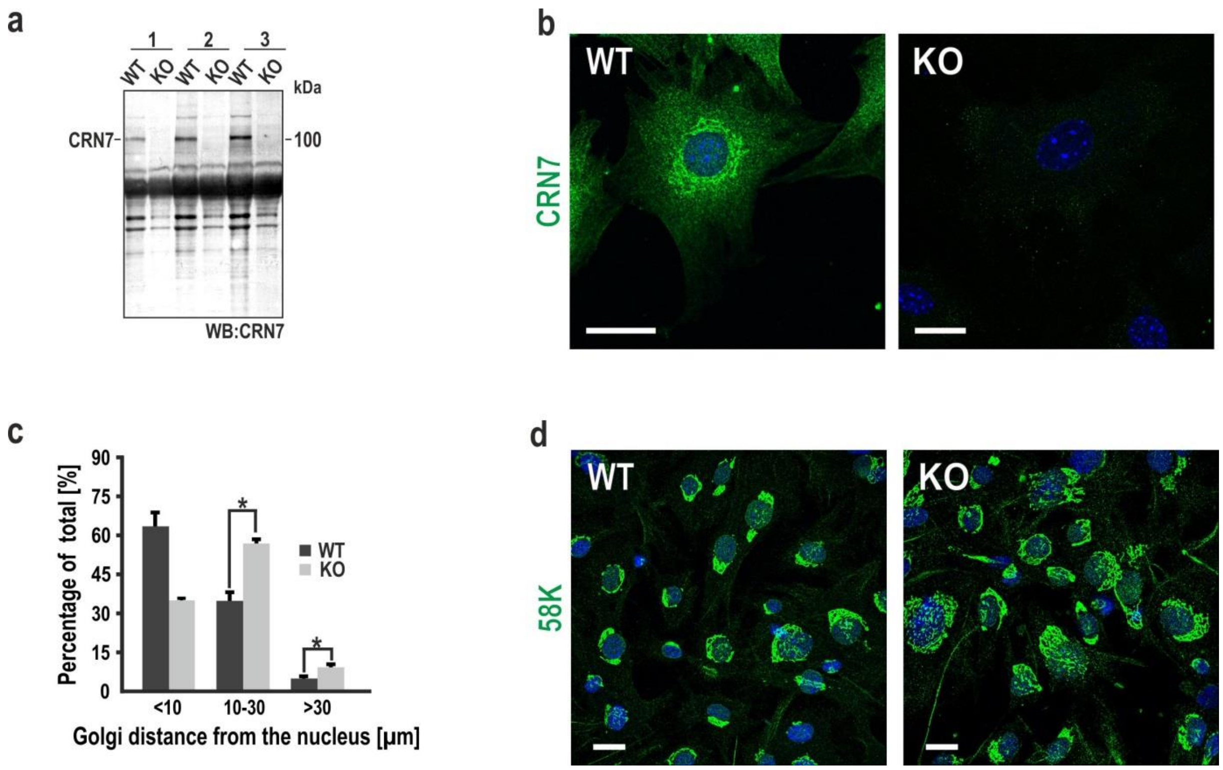
a
1
2
3
WT
KO
WT
KO
WT
KO
kDa
CRN7
100
WB:CRN7
b
WT
KO
CRN7
c
Percentage of total [%]
90
75
60
45
30
15
0
WT
KO
<10
10-30
>30
Golgi distance from the nucleus [μm]
d
WT
KO
58K

Figure S2

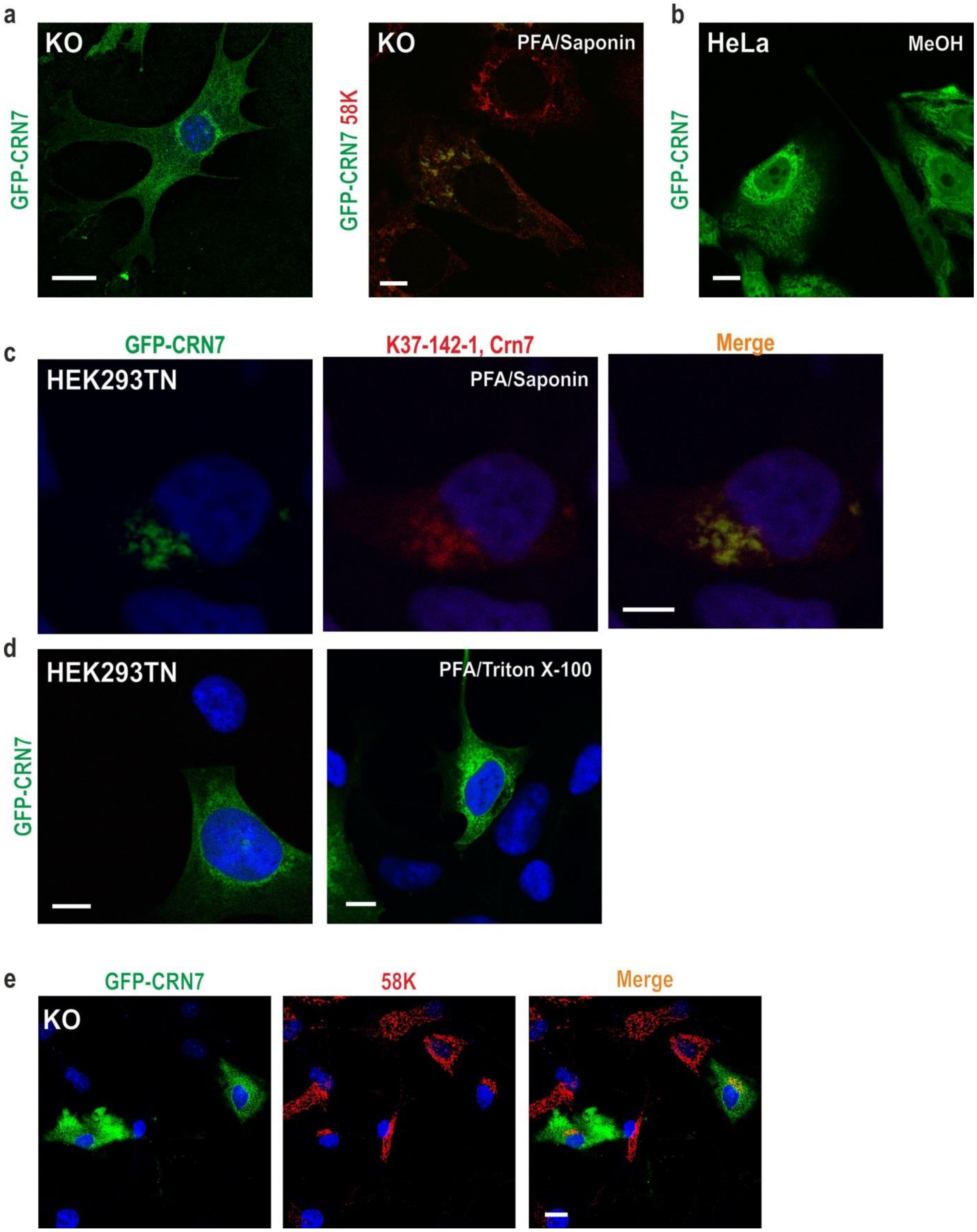
a
KO
GFP-CRN7
KO
PFA/Saponin
GFP-CRN7 58K
b
HeLa
MeOH
GFP-CRN7
c
GFP-CRN7
K37-142-1, Crn7
Merge
HEK293TN
PFA/Saponin
d
HEK293TN
PFA/Triton X-100
GFP-CRN7
e
GFP-CRN7
58K
Merge
KO

Figure S3

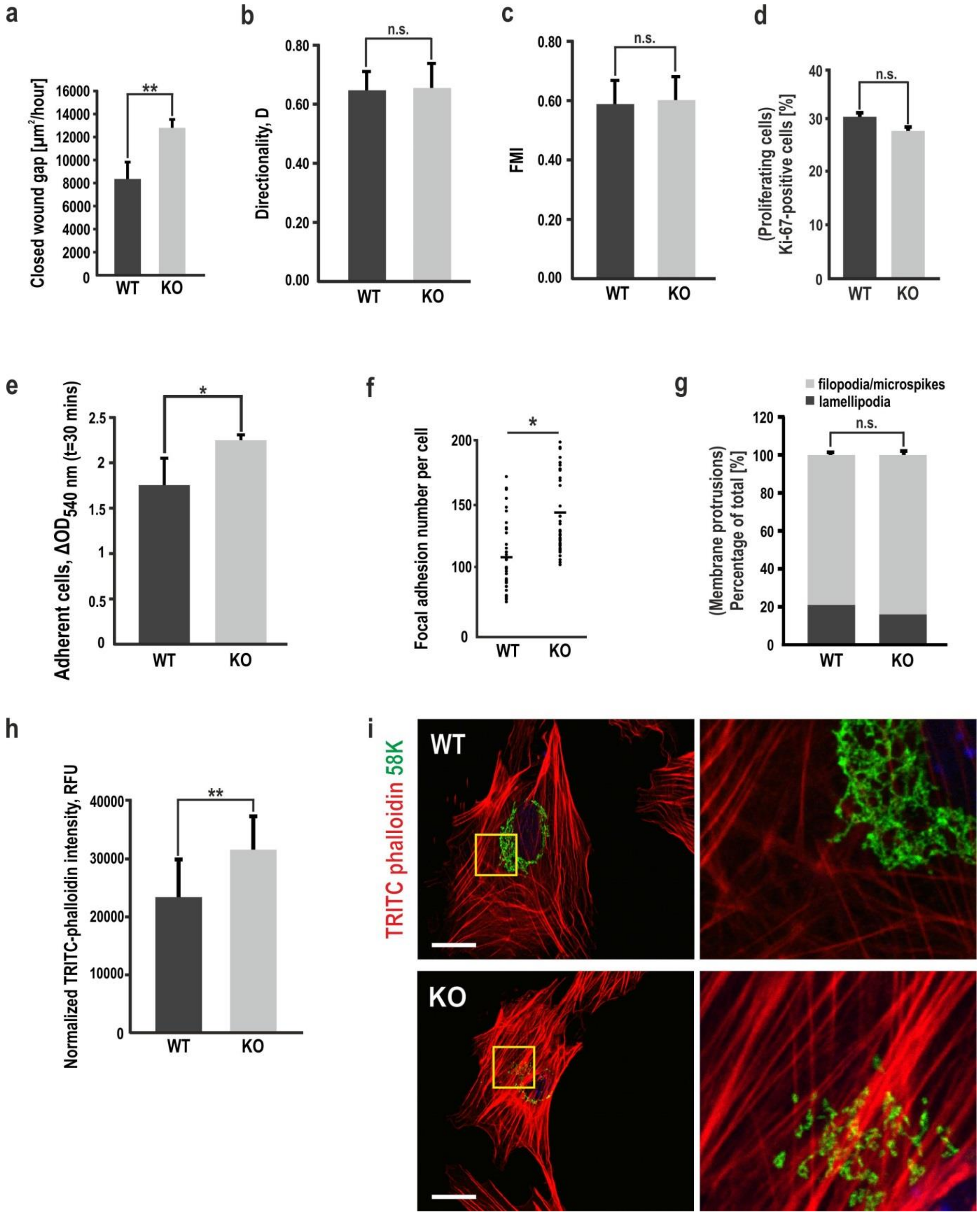
a
Closed wound gap [µm²/hour]
16000
14000
12000
10000
8000
6000
4000
2000
0
**
WT
KO
b
Directionality, D
0.80
0.60
0.40
0.20
0.00
n.s.
WT
KO
c
FMI
0.80
0.60
0.40
0.20
0.00
n.s.
WT
KO
d
(Proliferating cells)
Ki-67-positive cells [%]
40
30
20
10
0
n.s.
WT
KO
e
Adherent cells, ΔOD540 nm (t=30 mins)
2.5
2
1.5
1
0.5
0
*
WT
KO
f
Focal adhesion number per cell
200
150
100
0
*
WT
KO
g
filopodia/microspikes
lamellipodia
(Membrane protrusions)
Percentage of total [%]
120
100
80
60
40
20
0
n.s.
WT
KO
h
Normalized TRITC-phalloidin intensity, RFU
40000
30000
20000
10000
0
**
WT
KO
i
TRITC phalloidin 58K
WT
KO

Figure S4

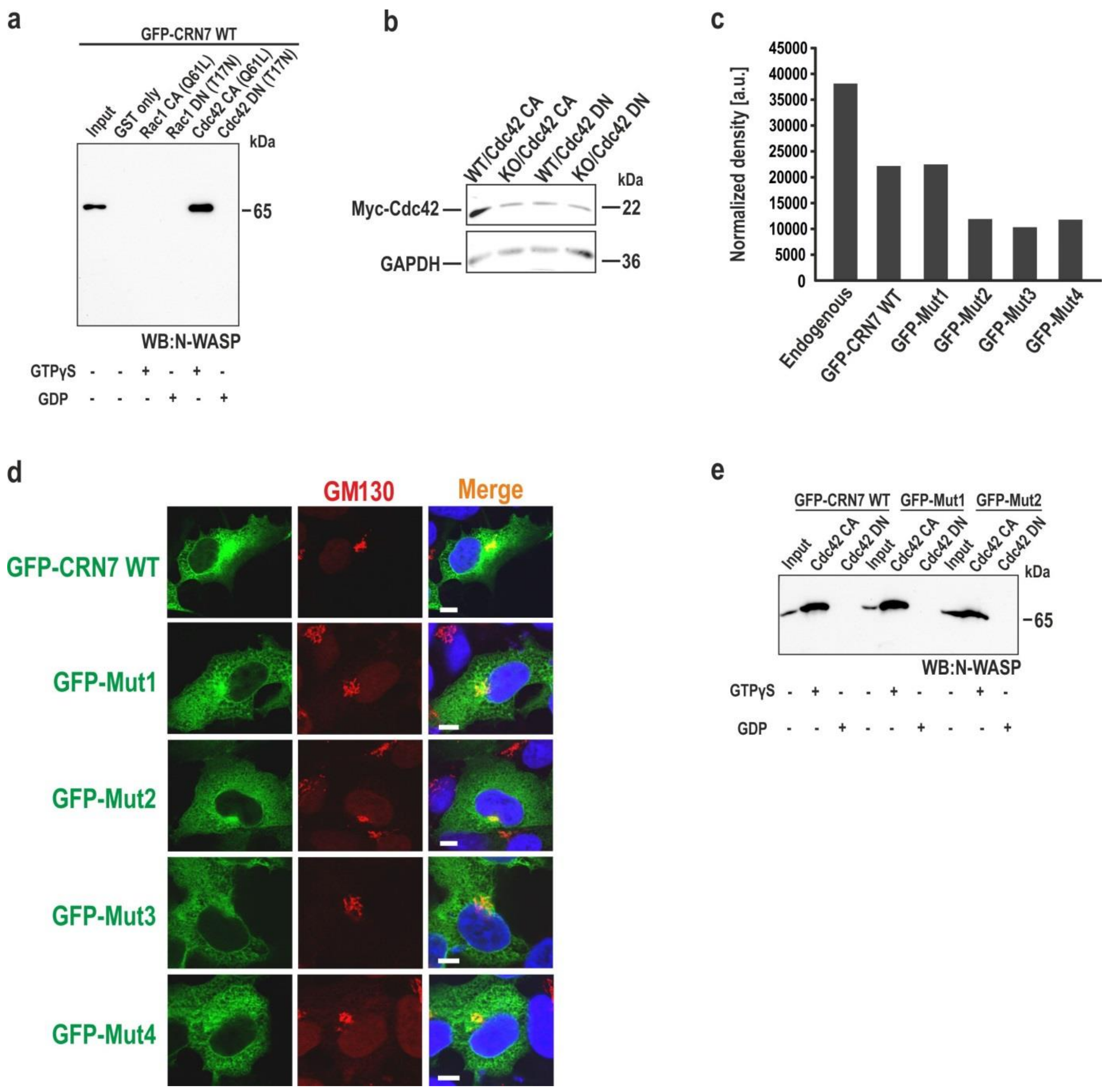

a
GFP-CRN7 WT
Input
GST only
Rac1 CA (Q61L)
Rac1 DN (T17N)
Cdc42 CA (Q61L)
Cdc42 DN (T17N)
kDa
65
WB:N-WASP
GTPγS - - + - + -
GDP - - - + - +
b
WT/Cdc42 CA
KO/Cdc42 CA
WT/Cdc42 DN
KO/Cdc42 DN
kDa
Myc-Cdc42
22
GAPDH
36
c
Normalized density [a.u.]
45000
40000
35000
30000
25000
20000
15000
10000
5000
0
Endogenous
GFP-CRN7 WT
GFP-Mut1
GFP-Mut2
GFP-Mut3
GFP-Mut4
d
GM130
Merge
GFP-CRN7 WT
GFP-Mut1
GFP-Mut2
GFP-Mut3
GFP-Mut4
e
GFP-CRN7 WT
GFP-Mut1
GFP-Mut2
Input
Cdc42 CA
Cdc42 DN
kDa
65
WB:N-WASP
GTPγS - + - - + - - + -
GDP - - + - - + - - +

Figure S5

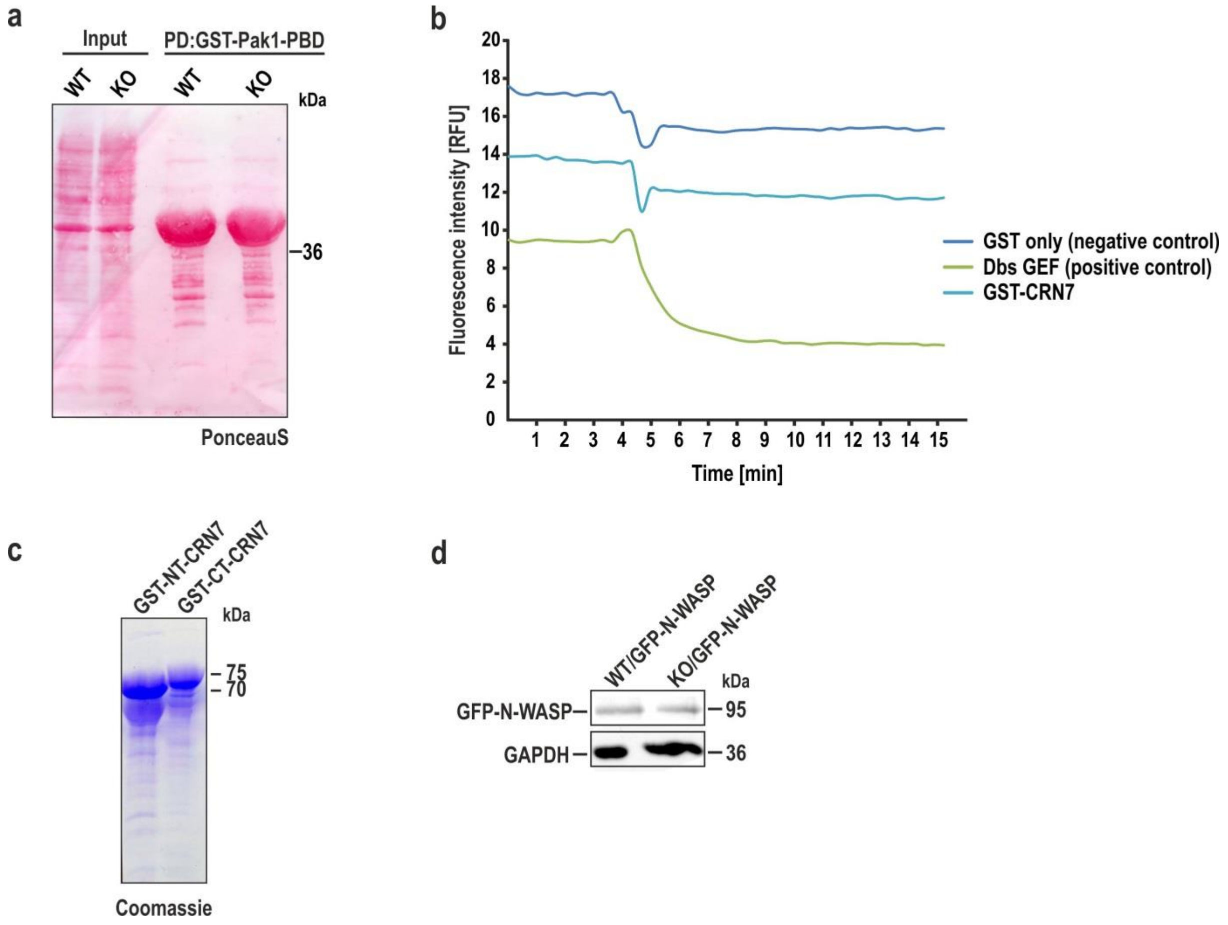
a
Input
PD:GST-Pak1-PBD
WT
KO
WT
KO
kDa
36
PonceauS
b
Fluorescence intensity [RFU]
Time [min]
GST only (negative control)
Dbs GEF (positive control)
GST-CRN7
c
GST-NT-CRN7
GST-CT-CRN7
kDa
75
70
Coomassie
d
WT/GFP-N-WASP
KO/GFP-N-WASP
kDa
GFP-N-WASP
95
GAPDH
36

Figure S6

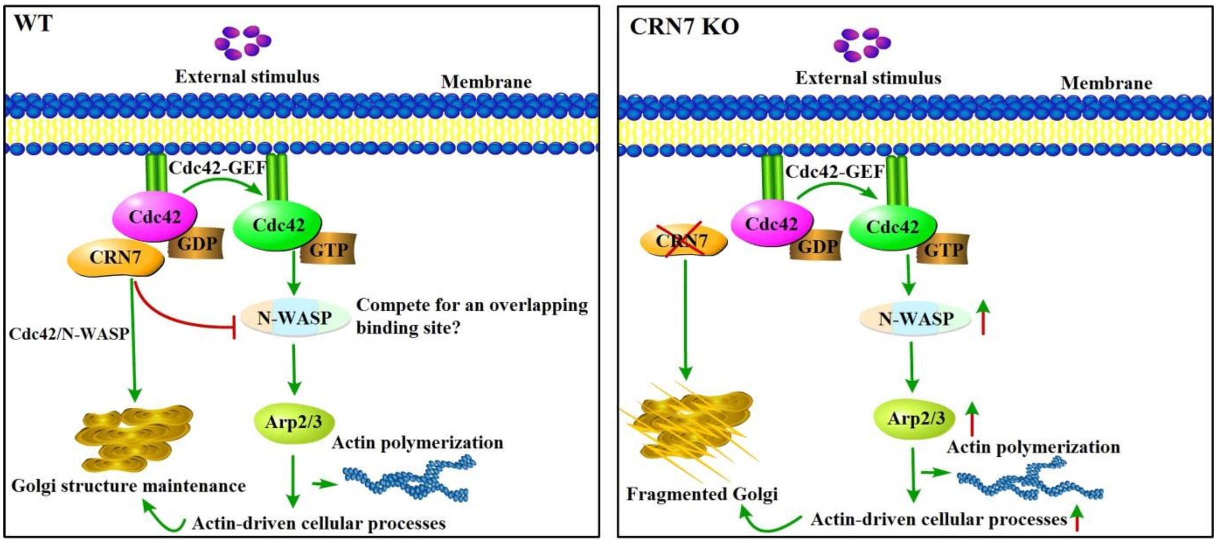
WT
External stimulus
Membrane
Cdc42-GEF
Cdc42
GDP
Cdc42
GTP
CRN7
N-WASP
Compete for an overlapping binding site?
Cdc42/N-WASP
Arp2/3
Actin polymerization
Golgi structure maintenance
Actin-driven cellular processes
CRN7 KO
External stimulus
Membrane
Cdc42-GEF
Cdc42
GDP
Cdc42
GTP
CRN7
N-WASP
Arp2/3
Actin polymerization
Fragmented Golgi
Actin-driven cellular processes

Table S1 Summary of the rescue potential of Cdc42 mutants in CRN7KO

Cellular Phenotypes	CRN7KO	KO/Myc-Cdc42CA	KO/Myc-Cdc42DN
**Cell spreading**	Enhanced compared to WT	Comparable to KO	Comparable to WT
**F-actin content**	Increased compared to WT	Comparable to KO	Comparable to WT
**Golgi morphology**	Fragmented unlike WT	Similar to KO	Partial rescue

Table S2 Summary of the rescue potential of CRN7-CRIB mutants

**Cellular Phenotypes**	**WT**	**CRN7KO**	**KO/GFP-CRN7WT**	**KO/GFP-Mut1**	**KO/GFP-Mut2**	**KO/GFP-Mut3**	**KO/GFP-Mut4**
**Golgi morphology**	Compact	Fragmented	Close to WT	Comparable to WT	Comparable to WT	Fails to rescue	Fails to rescue
**Cell polarization**	WT	Faster	-	-	-	-	-
**Single-cell motility**	WT	Accelerated	-	-	-	-	-
**Cell adhesion**	WT	Enhanced	-	-	-	-	-
**Cell spreading**	WT	Enhanced	Comparable to WT	Comparable to WT	Comparable to WT	Comparable to KO	Comparable to KO
**Cell proliferation**	WT	Like WT	-	-	-	-	-
**Membrane protrusion**	WT	Like WT	-	-	-	-	-
**F-actin content**	WT	Increased	Similar to WT	Comparable to WT	Comparable to WT	Partial rescue	Comparable to KO

* - not determined

Table S3 Summary of the rescue potential of full-length N-WASP in CRN7KO

**Cellular Phenotypes**	**CRN7KO**	**KO/GFP-N-WASP**
**Cell spreading**	Enhanced compared to WT	Partial rescue
**F-actin content**	Increased compared to WT	Partial rescue
**Golgi morphology**	Fragmented unlike WT	Fails to rescue

Materials and Methods

Constructs, Oligonucleotides and Antibodies

pEGFP-C1 and C2 (Clontech) and pGEX-4T-1 GST (GE Healthcare) were used for cloning and expression of CRN7. EGFP-GalT was obtained as a gift from Jennifer Lippincott-Schwartz (Addgene plasmid #11929). pRK5-Myc Cdc42 (Q61L and T17N), Rac1 (Q61L and T17N), pGEX-2T GST Cdc42 (WT, Q61L and T17N), Rac1 (Q61L and T17N), and pEGFP-C1 N-WASP full-length and deletion constructs [1] were obtained from Drs. Klemens Rottner and Anika Steffen (Braunschweig, Germany), pGEX-6P-1 GST Pak1-PBD from Dr, Jain Faix (MHH, Germany). pEGFP-C2 CRN7 carried full-length CRN7, CRN7 deletion constructs were cloned into pGEX-4T-1. pEGFP-C2 CRIB mutant constructs (Mut1,2,3,4) were prepared using a site directed mutagenesis kit (Stratagene) with the following primers (Sigma):

Mut1-F
GGCGACTGCCAGGGCCTGGCCAGGCCCTGCCCGCAGCAGCCGGGGTGGTGCTGG
GCCCCGAGGACCTCCCAGTGG

Mut1-R
CACTGGGAGGTCCTCGGGGCCCAGCACCACCCCGGCTGCTGCGGGCAGGGCCTG
GCCAGGCCCTGGCAGTCGCC

Mut2-F
GGATCCGACTGTGGCGGGTACCCGCAGAGGGCCTGGAAGAGGTGGCCGCCACGG
CAGAGACTGTGCTCACAGGCCACACGGAGAAGATCTG

Mut2-R
GCAGATCTTCTCCGTGTGGCCTGTGAGCACAGTCTCTGCCGTGGCGGCCACCTCTT
CCAGGCCCTCTGCGGGTACCCGCCACAGTCGGATCC

Mut4-F
GGCCGCCACGGCAGAGACTGTGCTCACAGGCGCCACGGAGAAGGCCTGCTCCCT
GCGCTTCCACCCACTGGCAGCCAATGTGCTGGCC

Mut4-R
GGCCAGCACATTGGCTGCCAGTGGGTGGAAGCGCAGGGAGCAGGCCTTCTCCGT
GGCGCCTGTGAGCACAGTCTCTGCCGTGGCGGCC

The antibodies used for western blot and immunofluorescence were as follows: GFP, mouse mAb K3-184-2 [2], CRN7, mAb K37-142-1, Myc, mAb 9E10, GST, mAb K84-913-0 were prepared in our laboratory, Rabbit GFP, polyclonal, was obtained from Dr. M. Schleicher (München), 58K, mAb 58K-9, Pericentrin, rabbit polyclonal, and Ki-67, rabbit polyclonal, were from Abcam, Mouse mAb GAPDH coupled to peroxidase (POD) and N-WASP, rabbit pAbs were from Sigma, Cdc42, mouse mAb was from Santa Cruz Biotechnology, mouse mAb vinculin was from Sigma, and GM130, mouse mAb was from BD Biosciences. All secondary antibodies were procured from Invitrogen.

Isolation of genomic DNA and PCR genotyping

Mouse tail tips were incubated overnight in lysis buffer (50 mM Tris-HCl, pH 8.5, 100 mM EDTA, 1% SDS, 100 mM NaCl) including proteinase K at 55°C. The DNA was precipitated with isopropanol, pelleted by centrifugation and washed with 70% ethanol. The DNA was solubilized with TE (Tris-EDTA, 10 and 1 mM, pH 8.0) buffer and stored at 4°C. To achieve a better solution of the DNA, the sample was incubated at 65°C prior to storage. The gDNA was used for genotyping using the following primers (Metabion):

3' Arm Specific (Ex4-F, P1)	AAGAGCTGCCTATGACCCAC
3' Gene Specific (GR-7, P2)	CCAACTCAGGAAACATAGGAC

Southern blotting

AseI digested genomic DNA was precipitated. The DNA fragments were separated by agarose gel electrophoresis (0.7% agarose) in 1X TAE (Tris base-Acetic acid-EDTA) buffer. Prior to transfer to nylon membranes (Biodyne 0.45 µm, Pall Corporation) the DNA was depurinated with 0.25 M HCl for 10-20 min. Crosslinking of the DNA to the nylon-membrane was performed with the UV Cross linker UVC 500 (Hoefer). For pre-hybridization membranes were placed in an incubation tube filled with pre-warmed Church buffer (7%

SDS, 0.5 M $NaPO_4$, 1 mM EDTA) and 100 µg/ml herring sperm DNA and incubated in a rotator for 1-2 h at 65°C. 25-50 ng of the double stranded probe (Neo probe) designed using primer pair: Neo-F CGCGGATCCGCCACCATGATTGAACAAGATGGATTGCACGC and Neo-R AACTGCAGAACCAATGCATTCAGAAGAACTCGTCAAGAAGGC was then denatured. For random priming a dCTP, dGTP and dTTP mixture (0.5 mM each), a hexanucleotide mixture, alpha ^{32}P labelled dATP and Klenow enzyme (2 U/µl) were added to the single stranded probe. Free nucleotides were removed by Sephadex G50 column chromatography (spun column). Hybridization was carried out at 65°C overnight on a rotator. After appropriate washing steps a film was exposed to the membrane and developed after 2-10 days of exposure at -80°C depending on the signal intensity.

RNA isolation, reverse transcriptase (RT) PCR and northern blotting

Total RNA was extracted from primary fibroblasts using Trizol reagent and following the instruction manual (Invitrogen). The concentration and quality of the total RNA was determined on a NanoDrop Agilent Bioanalyzer.

cDNA was prepared by reverse transcription of 2 µg RNA using random primers (pdN6 50 µM) and M-MLV-RT (200 U/µl, Promega). Primers were designed as shown below such that they span across two neighbouring exons to avoid genomic DNA contamination. The housekeeping gene GAPDH was used as a positive control, to ensure comparable concentrations of cDNA in the samples.

GAPDH-F	ACCACAGTCCATGCCATCAC
GAPDH-R	TCCACCACCCTGTTGCTGTA
Ex1-F	AAGTTTCGGCATATGGAGGC
Ex3-R	CTGAATGACAGCCAAGGTAG
Ex6-F	GTTGGAGGCCCACAAGGAC
Ex16-R	GTTAGCGCAGAAGCCATCAC

For northern blot analysis 10-20 µg of total RNA was separated on an agarose gel (1.2% agarose) in the presence of formaldehyde. The remaining steps of blotting (transfer from gel to membrane, labelling of probes, hybridization and washing) were same as that of Southern blotting described above.

Preparation of tissue or cell homogenates and protein quantitation

Frozen tissues (10 mg) were mixed with 1x SDS-sample buffer. The samples were heated at 95°C for 5 min. Afterwards samples were sonicated and centrifuged at room temperature. The supernatant was used for protein quantitation, SDS-PAGE and western blot analysis. For cell lysate preparation, for simply checking expression levels of proteins, cells washed with PBS were lysed with 1x SDS-sample buffer, sonicated, centrifuged and the cleared supernatant collected for analysis. The protein concentration was determined according to the protocol of the ProStain Protein Quantification Kit. Afterwards the samples were diluted with 1x SDS-sample buffer to achieve equal protein concentration and loaded on a gel.

Cell adhesion and proliferation assay

Equal numbers of cells were seeded in 96-well plates that were pre-coated with 20 µg/ml fibronectin and pre-blocked with 1% BSA, and incubated for 30 min at 37°C. 30 minutes post-plating the plates were shaken and rinsed to remove the detached cells. The attached cells were treated with 4% PFA and thereafter stained with Neutral Red (Sigma) solution, a dye which stains live cells only, and incubated at 37°C for 30 min. Following incubation, the stain was removed, wells rinsed and solubilisation solution (50% ethanol + 1% acetic acid) added to solubilise the dye from the stained cells and left to stand for 10 min with intermittent pipetting to enhance mixing of the solubilised dye. The absorbance was measured using a

TECAN plate-reader at a wavelength of 540 nm. The background absorbance of multi-well plates was measured at 690 nm and subtracted from 540 nm measurement as described in [3].

Cell proliferation was quantified from cells of equal density which were allowed to proliferate for 24 h, and were then stained for the proliferation marker Ki-67 (rabbit polyclonal antibodies, Abcam) for visual counting under the microscope.

Western blotting

Proteins were transferred from polyacrylamide gels onto nitrocellulose membranes by wet blotting or semi-dry method. The transfer was confirmed by PonceauS staining of the membrane. Blocking was performed with 5% milk powder in TBS-T (50 mM Tris, 150 mM NaCl, 0.05% Tween 20 adjusted to pH 7.6). For protein detection, the membrane was incubated with primary antibodies (purified or hybridoma supernatant) diluted in TBS-T for 1-2 h at room temperature or overnight at 4°C. After washes the membrane was incubated with the corresponding secondary peroxidase-conjugated antibodies. The membranes were incubated with the detection reagent (ChemiGlowTM, Alpha Innotech Corporation) and chemiluminescence signals were monitored with the FlourChemTMSP imaging system (Alpha Innotech) or by classical photographic film exposure. Antibodies were removed from nitrocellulose membranes by stripping of the membranes with 0.1 M NaOH for 10 minutes followed by washing steps with TBST.

Nucleotide exchange assay

For GDP release assay, 5 µM Cdc42WT-mantGDP (Jena Bioscience) was added to the reaction buffer (150 µl containing 100 mM NaCl, 50 mM Tris/HCl pH 7.4, 5 mM $MgCl_2$ and 2 mM beta-mercaptoethanol) containing molar excess of unlabelled GDP (Sigma). The dissociation of mant-GDP was monitored upon addition of 10 µM purified GST only, GST-

CRN7 and Dbs GEF by using the fluorimeter (Perkin-Elmer) settings: λ_{ex} = 290 nm, λ_{em} = 460 nm and slits = 5/5 at 25°C. The fluorescence emission was monitored over 15 min.

References

1. Benesch, S. et al. Phosphatidylinositol 4,5-biphosphate (PIP2)-induced vesicle movement depends on N-WASP and involves Nck, WIP, and Grb2. *J. Biol. Chem.* 277, 37771-37776 (2002).
2. Noegel, A.A. et al. The cyclase-associated protein CAP as regulator of cell polarity and cAMP signaling in *Dictyostelium*. *Mol. Biol. Cell* 15, 934-945 (2004).
3. Löwik, C.W., Alblas, M.J., van de Ruit, M., Papapoulos, S.E. & van der Pluijm, G. Quantification of adherent and nonadherent cells cultured in 96-well plates using the supravital stain neutral red. *Anal. Biochem.* 213, 426–433 (1993).
